# Calcium-mediated mitochondrial fission and mitophagy drive glycolysis to facilitate arterivirus proliferation

**DOI:** 10.1371/journal.ppat.1012872

**Published:** 2025-01-13

**Authors:** Zhe Sun, Zicheng Ma, Wandi Cao, Chenlong Jiang, Lei Guo, Kesen Liu, Yanni Gao, Juan Bai, Jiang Pi, Ping Jiang, Xing Liu

**Affiliations:** 1 Key Laboratory of Animal Diseases Diagnostic and Immunology, Ministry of Agriculture, MOE International Joint Collaborative Research Laboratory for Animal Health & Food Safety, College of Veterinary Medicine, Nanjing Agricultural University, Nanjing, China; 2 Jiangsu Co-Innovation Center for Prevention and Control of Important Animal Infectious Diseases and Zoonoses, Yangzhou, China; 3 Institute of Laboratory Medicine, Guangdong Provincial Key Laboratory of Medical Molecular Diagnostics, School of Medical Technology, The First Dongguan Affiliated Hospital, Guangdong Medical University, Dongguan, China; University of Georgia, UNITED STATES OF AMERICA

## Abstract

Mitochondria, recognized as the “powerhouse” of cells, play a vital role in generating cellular energy through dynamic processes such as fission and fusion. Viruses have evolved mechanisms to hijack mitochondrial function for their survival and proliferation. Here, we report that infection with the swine arterivirus porcine reproductive and respiratory syndrome virus (PRRSV), manipulates mitochondria calcium ions (Ca^2+^) to induce mitochondrial fission and mitophagy, thereby reprogramming cellular energy metabolism to facilitate its own replication. Mechanistically, PRRSV-induced mitochondrial fission is caused by elevated levels of mitochondria Ca^2+^, derived from the endoplasmic reticulum (ER) through inositol 1,4,5-triphosphate receptor (IP3R)—voltage-dependent anion channel 1 (VDAC1)—mitochondrial calcium uniporter (MCU) channels. This process is associated with increased mitochondria-associated membranes (MAMs), mediated by the upregulated expression of sigma non-opioid intracellular receptor 1 (SIGMAR1). Elevated mitochondria Ca^2+^ further activates the Ca^2+^/CaM-dependent protein kinase kinase β (CaMKKβ)—AMP-activated protein kinase (AMPK)—dynamin-related protein 1 (DRP1) signaling pathway, which interacts with mitochondrial fission protein 1 (FIS1) and mitochondrial dynamics proteins of 49 kDa (MiD49) to promote mitochondrial fission. PRRSV infection, alongside mitochondrial fission, triggers mitophagy via the PTEN-induced putative kinase 1 (PINK1)-Parkin RBR E3 ubiquitin (Parkin) pathway, promoting cellular glycolysis and excessive lactate production to facilitate its own replication. This study reveals the mechanism by which mitochondrial Ca^2+^ regulates mitochondrial function during PRRSV infection, providing new insights into the interplay between the virus and host cell metabolism.

## Author summary

PRRSV poses a major threat to the global swine industry, and no effective vaccines or antiviral drugs have been developed. A deep understanding of the PRRSV replication mechanism is essential for devising effective strategies to control this disease. The present work presents a detailed map of PRRSV infection, focusing on mitochondrial Ca^2+^, mitochondrial morphology, and glucose metabolism. Experimental findings reveal that viral infection induces alterations in fission and mitophagy through the elevation of mitochondrial Ca^2+^ levels. Furthermore, the study characterizes the regulatory mechanism of Ca^2+^ within mitochondria, influencing mitochondrial morphology during PRRSV infection. These mitochondrial alterations, coupled with reprogramming of cellular glucose metabolism, support the replication of the virus within host cells. This study provides insights into the interplay among the virus, mitochondrial morphology, and host cell metabolism, offering promise for guiding future research and informing therapeutic interventions against PRRSV.

## Introduction

Mitochondrial dynamics, characterized by the processes of mitochondrial fission and fusion, play a critical role in maintaining mitochondrial homeostasis, with precise regulation in response to changes in cellular physiology [1]. Mitochondrial fusion is tightly regulated by the highly conserved GTPase protein family, predominantly mediated by mitochondrial fusion protein 1 (MFN1), mitochondrial fusion protein 2 (MFN2), and optic atrophy protein 1 (OPA1); while mitochondrial fission is mediated by DRP1 [2]. DRP1 directs mitochondrial fission by relocating from the cytoplasm to the outer mitochondrial membrane, with the aid of adaptor proteins such as FIS1 and mitochondrial fission factor (MFF) [3,4]. Upon engagement, DRP1 forms a ring-like structure on the outer membrane, causing membrane constriction through protein aggregation, resulting in a narrow neck and subsequent organelle separation into two distinct structures [5]. Throughout this process, the activity of DRP1 is tightly regulated by post-translational modifications such as phosphorylation and dephosphorylation [6–8]. Mitochondrial fusion and fission are also accompanied by the process of mitophagy, which selectively removes damaged mitochondria through pathways like PINK1/Parkin, BCL2 interacting protein-3 like (NIX/BNIP3L), FUN14 domain containing 1 (FUNDC1), and microtubule-associated proteins light chain 3 (LC3) [9–12]. Fusion, fission, and mitophagy are crucial for maintaining normal mitochondrial function.

Ca^2+^, an omnipresent second messenger, is crucial for regulating fundamental mitochondrial functions. It acts as a cofactor in ATP synthesis for cellular energy production; influences the mitochondrial membrane potential to ensure efficient electron transport chain function for cellular energy balance, contributes to mitochondrial fusion and fission to maintain healthy morphology and quantity; triggers apoptosis and programmed cell death through mitochondrial membrane permeability transition; and modulates enzyme activity, influencing cellular metabolic processes [13,14]. Proper Ca^2+^ regulation is essential for mitochondrial function and overall cell health, while imbalances can lead to dysfunction.

For productive infectious replication and transmission, viruses employ strategies to manipulate host cell structures and pathways, resulting in changes to intracellular endomembrane and mitochondrial dynamics. Numerous studies have demonstrated that viruses can promote sustained infection by manipulating the mitochondrial dynamics of host cells [15]. For instance, hepatitis C virus (HCV) disrupts mitochondrial dynamics, promoting fission and mitophagy for viral persistence [16]. The influenza virus modifies mitochondrial dynamics by inducing mitochondria elongation [17]. Additionally, some viruses adeptly exploit the Ca^2+^ signal to shape a tailored cellular environment that meets their specific needs. For example, influenza virus infection induces Ca^2+^ influx [18], facilitating endocytic uptake, while herpes simplex virus-1 (HSV-1) downregulates voltage-gated calcium channels on infected neuronal cells to evade host cell detection [19].

PRRSV is a positive-sense single-stranded RNA virus classified in the family Arteriviridae within the order Nidovirales [20]. This virus causes an acute and highly contagious disease, resulting in significant mortality among piglets and reproductive disorders in pregnant sows [21,22]. Our previous study documented that PRRSV infection disrupts Ca^2+^ homeostasis, subsequently activating CaMKII-mediated autophagy, which supports viral replication [23]. Furthermore, Zhang et al. demonstrated that PRRSV glycoprotein 5 (GP5) enhances mitochondrial Ca^2+^, resulting in increased mitochondrial reactive oxygen species (ROS) and autophagy. This process alleviates NLR family pyrin domain containing 3 (NLRP3) inflammasome activation, promoting optimal viral replication [24]. In this study, we elucidate a comprehensive Ca^2+^-mediated pathway whereby PRRSV infection induces mitochondrial fission and mitophagy, enhancing glycolytic metabolism and leading to the accumulation of excess lactate, ultimately facilitating viral replication.

## Results

### PRRSV infection triggers mitochondrial fission to promote its replication

**Fig 1:**
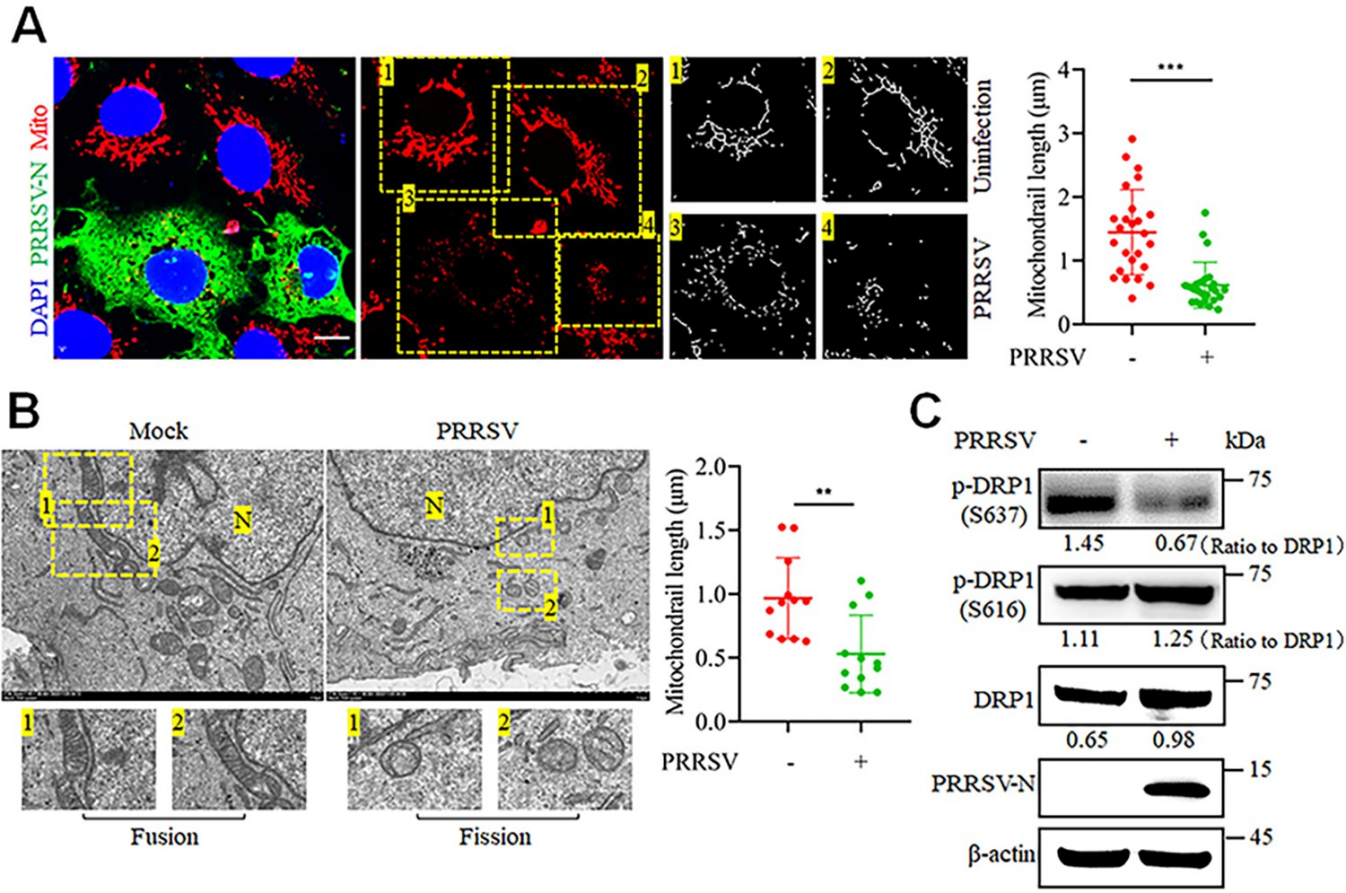
PRRSV infection triggers mitochondrial fission. (A-C) Marc-145 cells were infected with PRRSV (MOI = 0.1) for 24 h. (A) The cells were stained with MitoTracker Red, PRRSV-N antibody, and DAPI, followed by confocal microscopy. Enlarged images show the typical tubular mitochondrial network in uninfected cells and fragmented mitochondria in infected cells. Quantitative analysis of mitochondrial length is presented on the right. The lengths of mitochondria from 25 cells were quantified in each group. (B) The ultrastructure of mock-infected and PRRSV-infected cells was examined by electron microscopy. “N” represents nuclei. Zoomed-in images show normal elongated tubular mitochondria in uninfected cells and fragmented mitochondria with loss of cristae in infected cells. Scale bar = 2 μm. Quantification of mitochondrial length (n = 12) is shown on the right. (C) Mock-infected and PRRSV-infected cells were harvested for western blot analysis using antibodies against p-DRP1(S637), p-DRP1(S616), DRP1, PRRSV-N and β-actin. The levels of phosphorylated DRP1 were normalized to total DRP1 protein, while the levels of other proteins were normalized to β-actin. Data are expressed as means ± SD, n = 25 in A and n = 12 in B. *p<0.05; **p < 0.01; ***p < 0.001. The data are representative of results from three independent experiments.

To investigate whether PRRSV infection can alter cellular mitochondrial dynamics, Marc-145 cells were mock-infected or infected with PRRSV for 24 hours. Confocal microscopy revealed that PRRSV-infected cells, in comparison to uninfected cells with typical tubular mitochondrial network structure, displayed evident mitochondrial fragmentation, indicating pronounced mitochondrial fission (Fig 1A). Ultrastructural analysis by electron microscopy confirmed significant swelling and shortening of the mitochondria, along with disruption of mitochondrial filamentous structures, in PRRSV-infected as compared to mock-infected cells (Fig 1B). For additional verification, we performed western blot analysis of DRP1, a crucial protein in mitochondrial fission that is known to be differentially regulated by phosphorylation [7,25]. Compared to samples from mock-infected cells, samples from PRRSV-infected cells had decreased phosphorylation at Ser637 and elevated total DRP1 and phosphorylation at Ser616 (Fig 1C).

**Fig 2:**
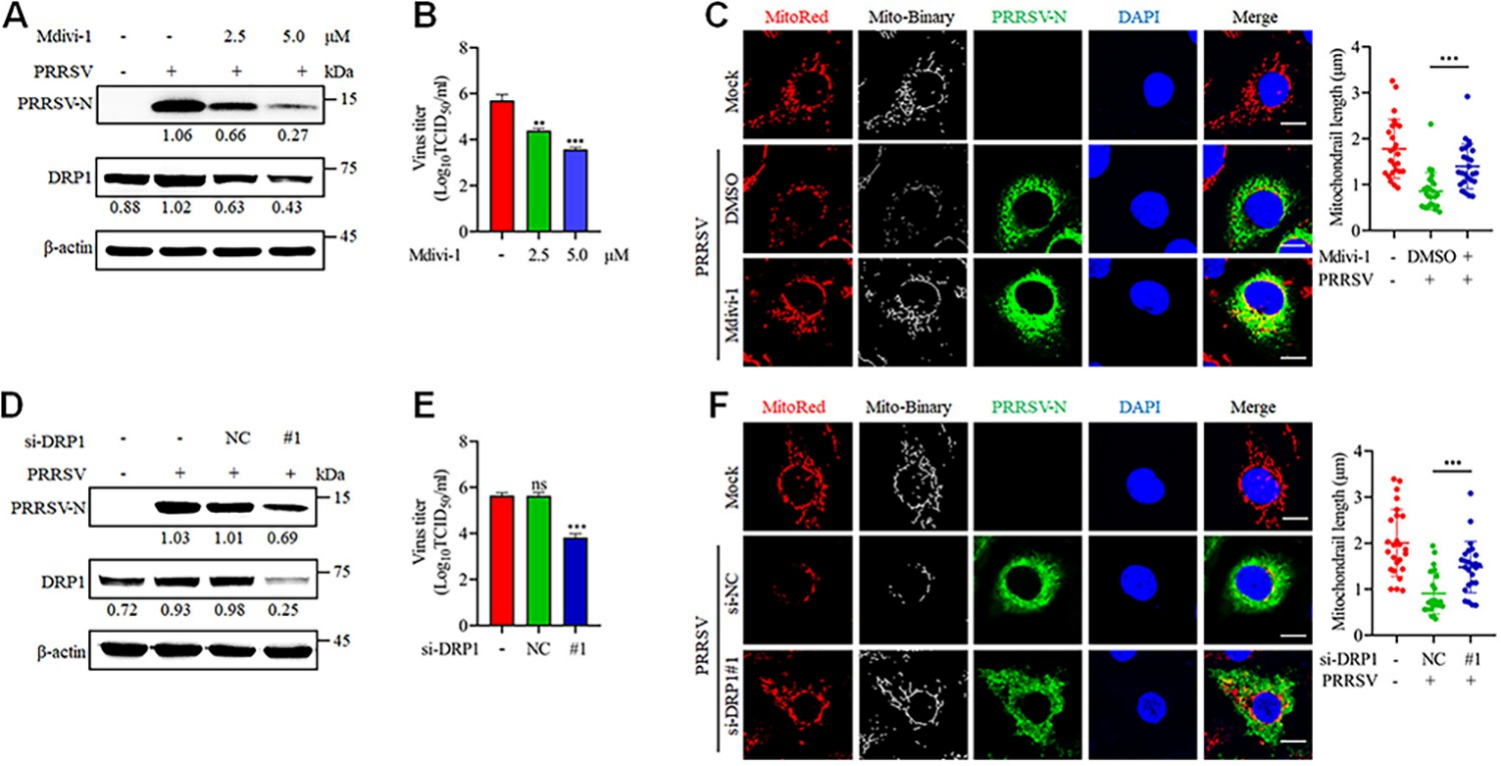
Mitochondrial fission facilitates PRRSV replication. (A-F) Marc-145 cells were mock-infected or infected with PRRSV (MOI = 0.1) for 24 h with the indicated doses of Mdivi-1 (A-C) or siRNA (D-F). (A and D) Cell lysates were harvested for western blot analysis with antibodies against PRRSV-N, DRP1 and β-actin. The levels of proteins were normalized to β-actin. (B and E) Determination of the TCID_50_ of PRRSV in the cell supernatant. (C and F) Marc-145 cells were incubated with MitoTracker Red, PRRSV-N antibody and DAPI, followed by observation under a confocal microscope. Quantitative analysis of mitochondrial length is presented on the right. The lengths of mitochondria from 25 cells per group were quantified. Data are expressed as means ± SD, n = 3 in B and E and n = 25 in C and F. *p<0.05; **p < 0.01; ***p < 0.001. The data are representative of results from three independent experiments.

Next, we determined the significance of mitochondrial fission for PRRSV replication using mitochondrial division inhibitor 1 (Mdivi-1), a specific inhibitor of DRP1. Mdivi-1 significantly suppressed both PRRSV-N protein expression and viral titers in a dose-dependent manner (Fig 2A and 2B). PRRSV-induced mitochondrial fission was also reversed by Mdivi-1 treatment (Fig 2C). To further corroborate the results, DRP1-targeting siRNAs were designed (S1B Fig). Consistent with the Mdivi-1 results, DRP1 knockdown led to a reduction in PRRSV-N protein expression and viral titers, along with the reversal of mitochondrial fragmentation (Fig 2D–2F). Neither Mdivi-1 nor siRNA treatments had any impact on cell viability, confirming their functional specificity (S1A and S1C Fig). Collectively, these results indicate that PRRSV-activated DRP1 regulates mitochondrial fission, and that mitochondrial fission enhances PRRSV replication.

### Mitochondrial Ca^2+^ influx through IP3R-VDAC1-MCU channels regulates PRRSV-induced mitochondrial fission

Our previous study demonstrated that PRRSV infection promotes the excessive accumulation of extracellular Ca^2+^ in the ER, which is subsequently released into the cytoplasm through the IP3R channel [23]. We hypothesized that some of Ca^2+^ from the ER may transfer to the mitochondria, thereby inducing mitochondrial fission during PRRSV infection. To this end, we assessed alterations in mitochondrial Ca^2+^ levels following PRRSV infection. Rhod-2 staining, followed by both confocal microscopy and flow cytometry analyses revealed a substantial increase in mitochondrial Ca^2+^ induced by PRRSV infection (S2A and S2B Fig).

**Fig 3:**
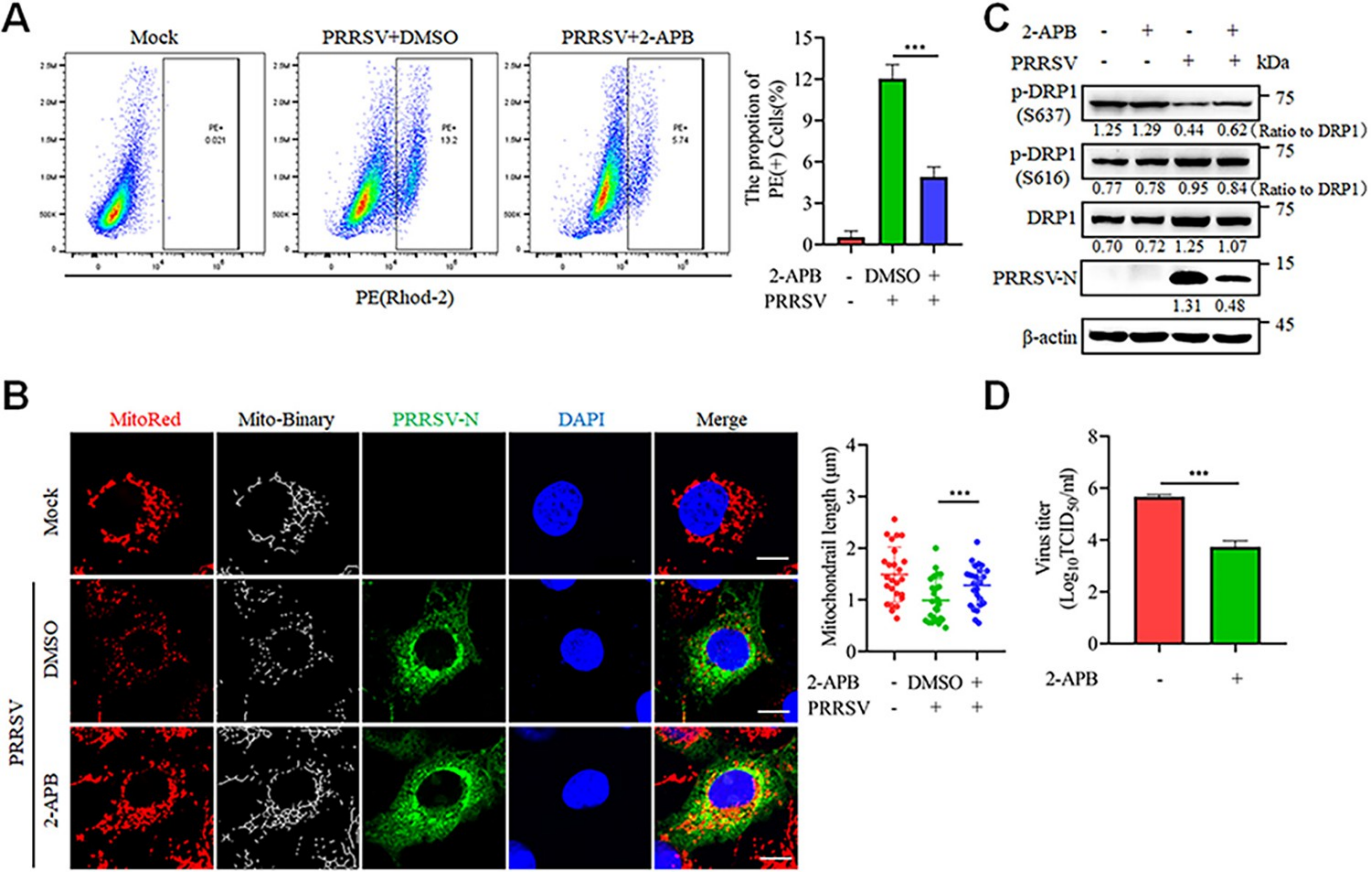
Mitochondrial Ca^2+^ from IP3R channel mediates mitochondrial fission. (A-D) Marc-145 cells were infected with PRRSV (MOI = 0.1) for 24 h with or without 2-APB treatment. (A) Ca^2+^ was detected by flow cytometry after Rhod-2 staining. (B) The cells were analyzed by confocal microscopy after staining with MitoTracker Red, PRRSV-N antibody and DAPI. The mitochondrial length was quantified from 25 cells per group (right panel). (C) The cell lysates were harvested for western blot analysis with antibodies against p-DRP1(S637), p-DRP1(S616), DRP1, PRRSV-N and β-actin. The levels of phosphorylated DRP1 were normalized to the total DRP1 protein, while the levels of other proteins were normalized to β-actin. (D) Quantification of the TCID_50_ of PRRSV in cell supernatants. Data are expressed as means ± SD, n = 3 in A and D or n = 25 in B. *p<0.05; **p < 0.01; ***p < 0.001. The data are representative of results from three independent experiments.

Next, we utilized the IP3R-specific inhibitor, 2-Aminoethyl Diphenylborinate (2-APB) to investigate whether ER Ca^2+^ is released into the mitochondria via the IP3R channel following PRRSV infection. The application of 2-APB alone did not significantly alter mitochondrial Ca^2+^ levels or cell viability (S2C and S2D Fig). However, 2-APB markedly reduced mitochondrial Ca^2+^ levels upon PRRSV infection (Fig 3A). Furthermore, 2-APB prevented mitochondrial fission, partially reversed the decrease in DRP1 Ser637 phosphorylation and the increase in DRP1 Ser616 phosphorylation, and inhibited PRRSV replication (Fig 3B–3D).

**Fig 4:**
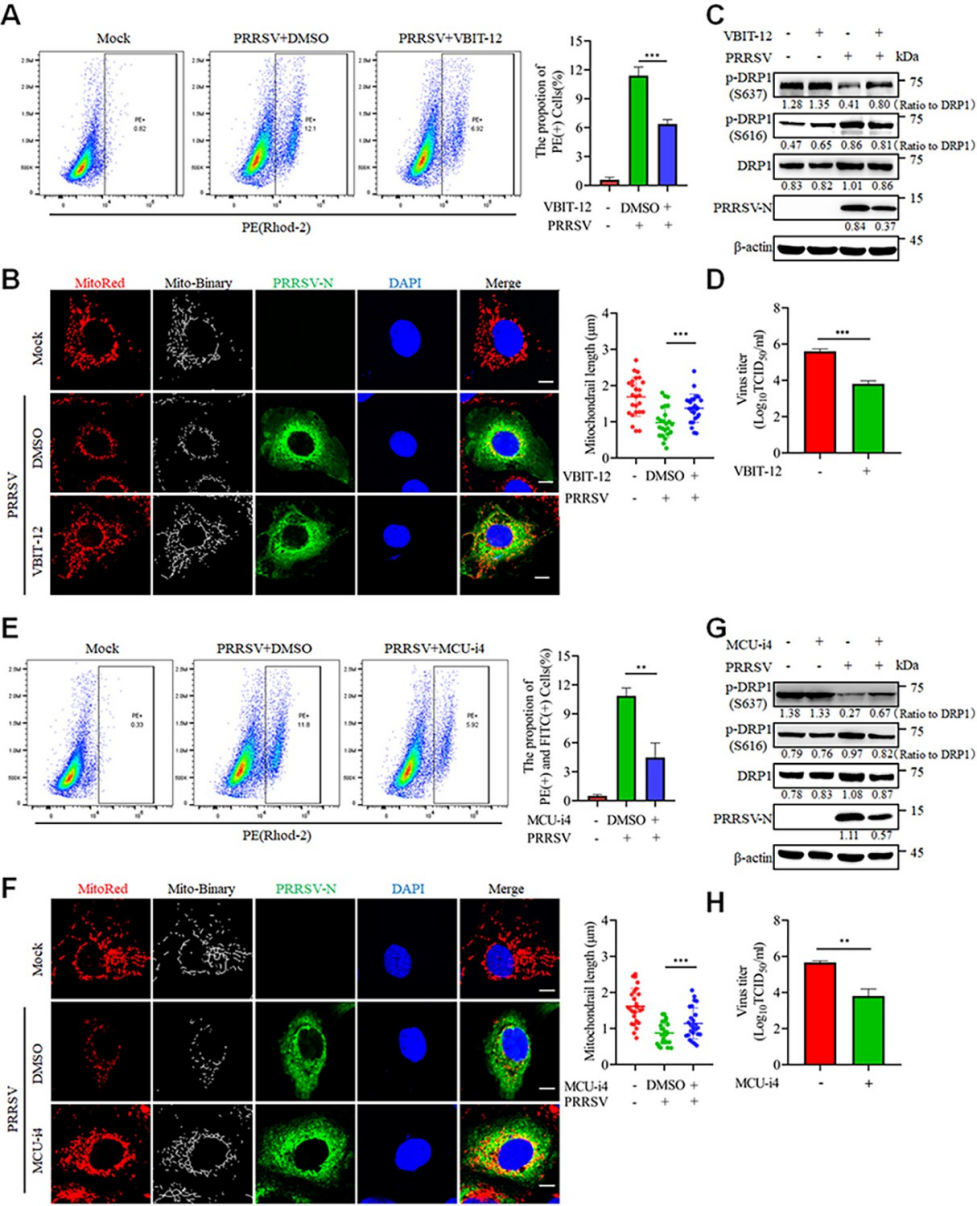
VDAC1-MCU channels mediate mitochondrial Ca^2+^ entry and mitochondrial fission. (A-H) Marc-145 cells were infected with PRRSV (MOI = 0.1) for 24 h with or without VBIT-12 (A-D) or MCU-i4 (E-H) treatment. (A and E) Ca^2+^ was detected by flow cytometry after Rhod-2 staining. (B and F) The cells were analyzed by confocal microscopy after staining with MitoTracker Red, PRRSV-N antibody and DAPI. The mitochondrial length was quantified from 25 cells per group (right panel). (C and G) The cell lysates were harvested for western blot analysis with antibodies against p-DRP1(S637), p-DRP1(S616), DRP1, PRRSV-N and β-actin. The levels of phosphorylated DRP1 were normalized to the total DRP1 protein, while the levels of other proteins were normalized to β-actin. (D and H) Quantification of the TCID_50_ of PRRSV in cell supernatants. Data are expressed as means ± SD, n = 3 in A, D, E and H or n = 25 in B and F. *p<0.05; **p < 0.01; ***p < 0.001. The data are representative of results from three independent experiments.

The uptake of Ca^2+^ into the mitochondria is primarily facilitated through the entry channels VDAC1 and MCU [26]. Therefore, we utilized specific inhibitors of VDAC1 and MCU (VBIT-12 and MCU-i4) to investigate whether VDAC1 and MCU participate in PRRSV-mediated mitochondrial Ca^2+^ influx. Similarly, neither VBIT-12 nor MCU-i4 alone affected mitochondrial Ca^2+^ levels or exhibited cytotoxicity (S2E–S2H Fig), but both treatments reduced mitochondrial Ca^2+^ levels upon PRRSV infection (Fig 4A and 4E). Consistently, both VBIT-12 and MCU-i4 inhibited mitochondrial fission, restored the changed phosphorylation levels of DRP1 Ser637 and DRP1 Ser616, and reduced PRRSV titers (Fig 4B–4D and Fig 4F–4H). These results suggest that PRRSV utilizes the IP3R-VDAC1-MCU channels to transport Ca^2+^ from the ER to the mitochondria, resulting in overloaded mitochondrial Ca^2+^, mitochondrial fission, and viral replication.

### Increased formation of MAMs mediated by SIGMAR1 facilitates the uptake of mitochondrial Ca^2+^

**Fig 5:**
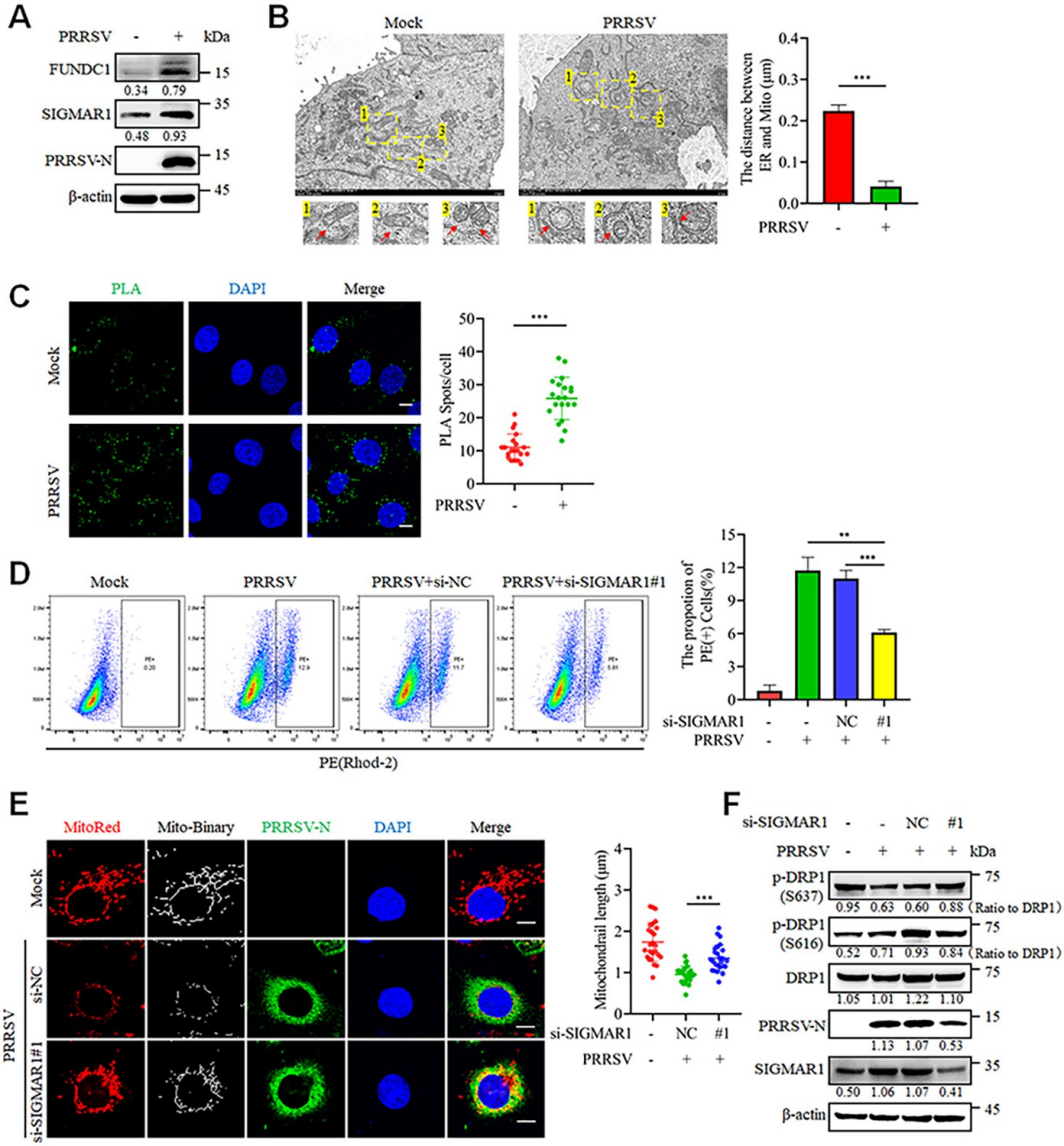
PRRSV infection increases mitochondria-ER contacts mediated by SIGMAR1. (A-C) Marc-145 cells were infected with PRRSV (MOI = 0.1) for 24 h. (A) The cell lysates were harvested for western blot analysis with antibodies against FUNDC1, SIGMAR1, PRRSV-N and β-actin. (B) Transmission electron microscopy was employed to observe the contact between the ER and mitochondria in mock-infected and PRRSV-infected cells. The mitochondrial-endoplasmic reticulum contacts were quantified using Image J (n = 20). (C) The cells were subjected to PLA with anti-Tom20 and anti-Calnexin antibodies. The number of PLA spots was quantified for 20 cells per group. (D-F) Marc-145 cells were transfected with control siRNA or siRNA targeting SIGMAR1 for 24 h prior to mock infection or infection with PRRSV (0.1 MOI) for an additional 24 hours. (D) Ca^2+^ levels were assessed using flow cytometry with Rhod-2 staining. (E) The mock-infected and PRRSV-infected cells were analyzed by confocal microscopy after staining with MitoTracker Red, PRRSV-N antibody and DAPI. The mitochondrial length was quantified from 25 cells per group (right panel). (F) The cell lysates were harvested for western blot analysis with antibodies against p-DRP1(S637), p-DRP1(S616), DRP1, PRRSV-N, SIGMAR1 and β-actin. The levels of phosphorylated DRP1 were normalized to the total DRP1 protein, while the levels of other proteins were normalized to β-actin. Data are expressed as means ± SD, n = 20 in B and C, n = 3 in D or n = 25 in E. *p<0.05; **p < 0.01; ***p < 0.001. The data are representative of results from three independent experiments.

The ER and mitochondria are closely interconnected at specific sites known as MAMs, which play a crucial role in Ca^2+^ exchange between the two organelles [27,28]. Therefore, we investigated the impact of PRRSV on the formation of MAMs. The results demonstrate that PRRSV infection significantly increased the expression of MAM component proteins FUNDC1 and SIGMAR1 (Fig 5A). Furthermore, ultrastructural analysis by transmission electron microscopy confirmed that the distance between the ER and mitochondria was reduced in PRRSV-infected cells compared to mock-infected cells (Fig 5B). To directly visualize the interaction between ER and mitochondria within cells, a proximity ligation assay (PLA)—an experimental tool that enables the visualization of in situ co-localizations between proteins less than 40 nm apart—was conducted. The results showed that PRRSV infection significantly increased the number of spots indicating interactions between Tom20 and Calnexin, suggesting enhanced contact between the mitochondria and the endoplasmic reticulum (Fig 5C). Because SIGMAR1 has been shown to modulate Ca^2+^ signaling between ER and mitochondria [29], we investigated the impact of SIGMAR1 siRNA on mitochondrial Ca^2+^. The results confirmed that SIGMAR1 knockdown significantly inhibited mitochondrial Ca^2+^ induced by PRRSV (Fig 5D), with no detectable effect on cell viability under the doses employed (S3 Fig). Furthermore, downregulation of SIGMAR1 expression reversed PRRSV-induced mitochondrial fission, as evaluated by confocal fluorescence staining experiments (Fig 5E); significantly restored the levels of DRP1 phosphorylation at Ser637 and Ser616; and reduced the PRRSV-N expression level (Fig 5F). Collectively, these results indicate that PRRSV promotes the formation of MAMs, and that the component protein SIGMAR1 enhances mitochondrial Ca^2+^ uptake during PRRSV infection.

### The CaMKKβ-AMPK-DRP1 signaling pathway orchestrates PRRSV-induced mitochondrial fission

**Fig 6:**
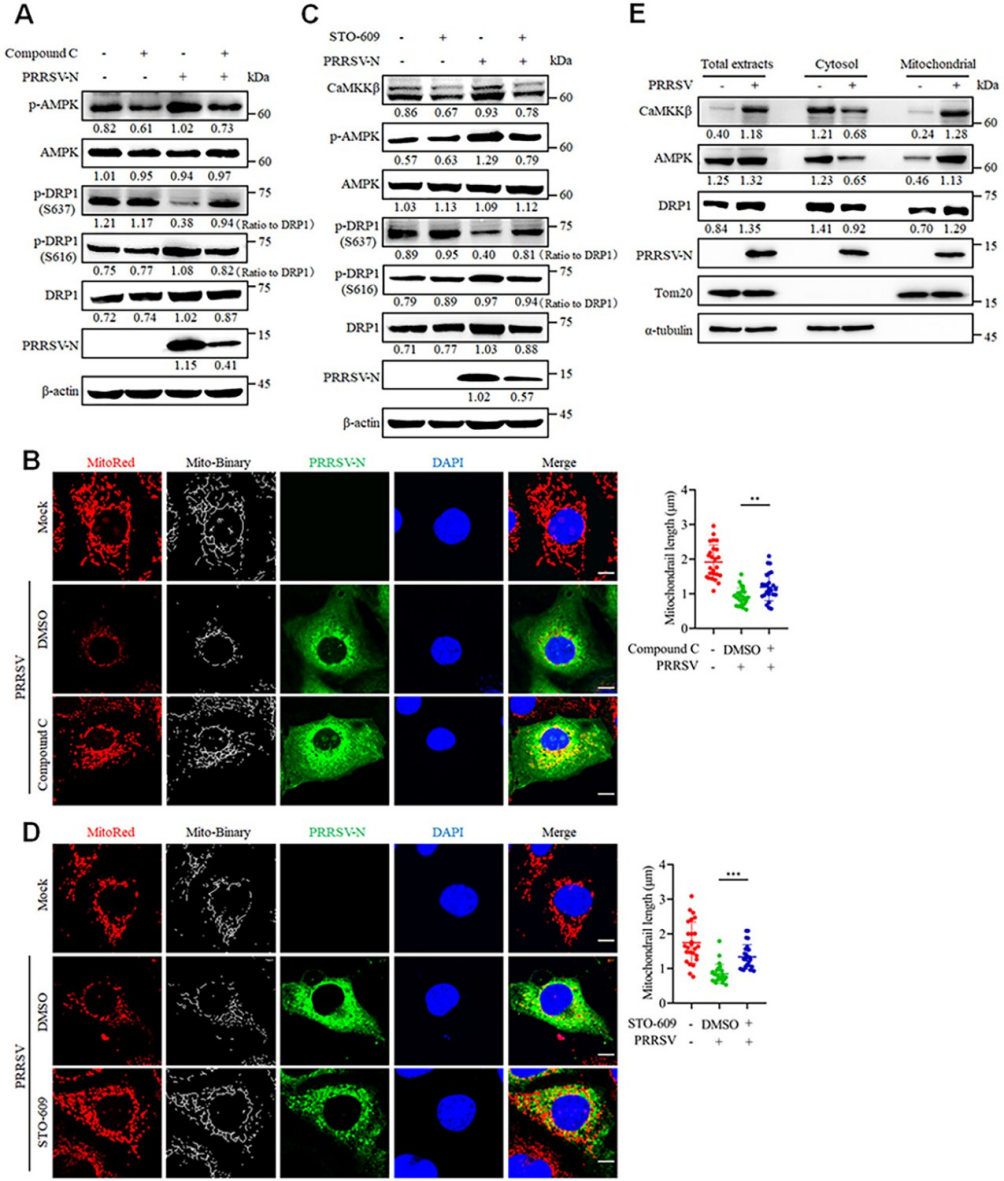
The CaMKKβ-AMPK-DRP1 cascade mediates PRRSV-induced mitochondrial fission. (A-D) Marc-145 cells were mock-infected or infected with PRRSV (MOI = 0.1), with or without Compound C (10 μM) or STO-609 (10 μM) treatment for 24 hours. (A and C) The cell lysates were collected and subjected to western blot analysis with antibodies against CaMKKβ (panel C only), p-AMPK, AMPK, p-DRP1 (S637), p-DRP1 (S616), DRP1, PRRSV-N and β-actin. The levels of phosphorylated DRP1 were normalized to the total DRP1 protein, while the levels of other proteins were normalized to β-actin. (B and D) The cells were stained with MitoTracker Red, PRRSV-N antibody and DAPI, followed by confocal microscopy. The mitochondrial length was quantified from 25 cells per group (right panel). (E) Marc-145 cells were mock-infected or infected with PRRSV (MOI = 0.1) for 24 h, and then harvested for cytoplasmic and mitochondrial fractionation. Samples were subjected to western blot analysis with antibody against CaMKKβ, AMPK, DRP1, PRRSV-N, Tom20 and α-tubulin. The levels of proteins were normalized to Tom20 or α-tubulin. Data are expressed as means ± SD, n = 25 in B and D. *p<0.05; **p < 0.01; ***p < 0.001. The data are representative of results from three independent experiments.

Next, we sought to explore the signaling cascades underlying changes in DRP1 phosphorylation in response to PRRSV infection. The synthesis of CaMKKβ is known to be facilitated by Ca^2+^, and its downstream factor AMPK has been shown to mediate changes in DRP1 phosphorylation [30,31]. Additionally, calcineurin (CaN), a serine/threonine phosphatase, is activated by Ca^2+^ and facilitates DRP1 dephosphorylation [32]. To evaluate whether the CaMKKβ-AMPK and/or CaN signaling pathways mediate changes in DRP1 phosphorylation upon PRRSV infection, the expression levels of these proteins were examined by western blotting. The results demonstrated that PRRSV infection markedly increased the expression of CaMKKβ, concurrently elevating the phosphorylated levels of AMPK, but had no impact on CaN expression (S4A Fig). To gain deeper insights into the roles of these signaling pathways, we evaluated the effects of specific inhibitors for CaMKKβ (i.e., STO-609), AMPK (i.e., Compound C), and CaN (i.e., FK506), each of which were confirmed to have no adverse effects on cell viability at the given doses (S4D–S4F Fig). The western blot results revealed that Compound C inhibited AMPK phosphorylation and simultaneously inhibited DRP1 Ser616 phosphorylation and restored DRP1 Ser637 dephosphorylation (Fig 6A). Furthermore, STO-609 effectively suppressed CaMKKβ expression, reduced downstream AMPK phosphorylation and DRP1 Ser616 phosphorylation, and restored PRRSV-induced DRP1 Ser637 dephosphorylation (Fig 6C). Compound C and STO-609 each also significantly simultaneously reversed PRRSV-induced mitochondrial fission (Fig 6B and 6D). However, administration of the CaN inhibitor FK506 had no impact on PRRSV-induced DRP1 phosphorylation or mitochondrial fission (S4B and S4C Fig). To rule out the non-specific effects of inhibitors, siRNA knockdown assays targeting CaMKKβ and AMPK were conducted to investigate their relationship with DRP1. The results showed that knockdown of CaMKKβ and AMPK produced effects similar to those observed with Compound C and STO-609 (S4G and S4H Fig). These results suggest that DRP1 phosphorylation changes in response to PRRSV infection are mediated by CaMKKβ-AMPK but not CaN.

Typically, DRP1 resides in the cytoplasm; however, when mitochondrial fission is activated, DRP1 translocates to the mitochondria and orchestrates the division process there [3,4]. Given the involvement of the CaMKKβ-AMPK axis in DRP1-mediated mitochondrial fission during PRRSV infection, we investigated the distribution of CaMKKβ, AMPK and DRP1 in the cytoplasm and mitochondria. As expected, PRRSV infection promoted the translocation of each of these proteins from the cytoplasm to the mitochondria (Fig 6E), thus providing additional support for the involvement of CaMKKβ-AMPK-DRP1 signaling in facilitating mitochondrial fission during PRRSV infection.

### The DRP1 adaptors FIS1 and MiD49 contribute to PRRSV-induced mitochondrial fission

**Fig 7:**
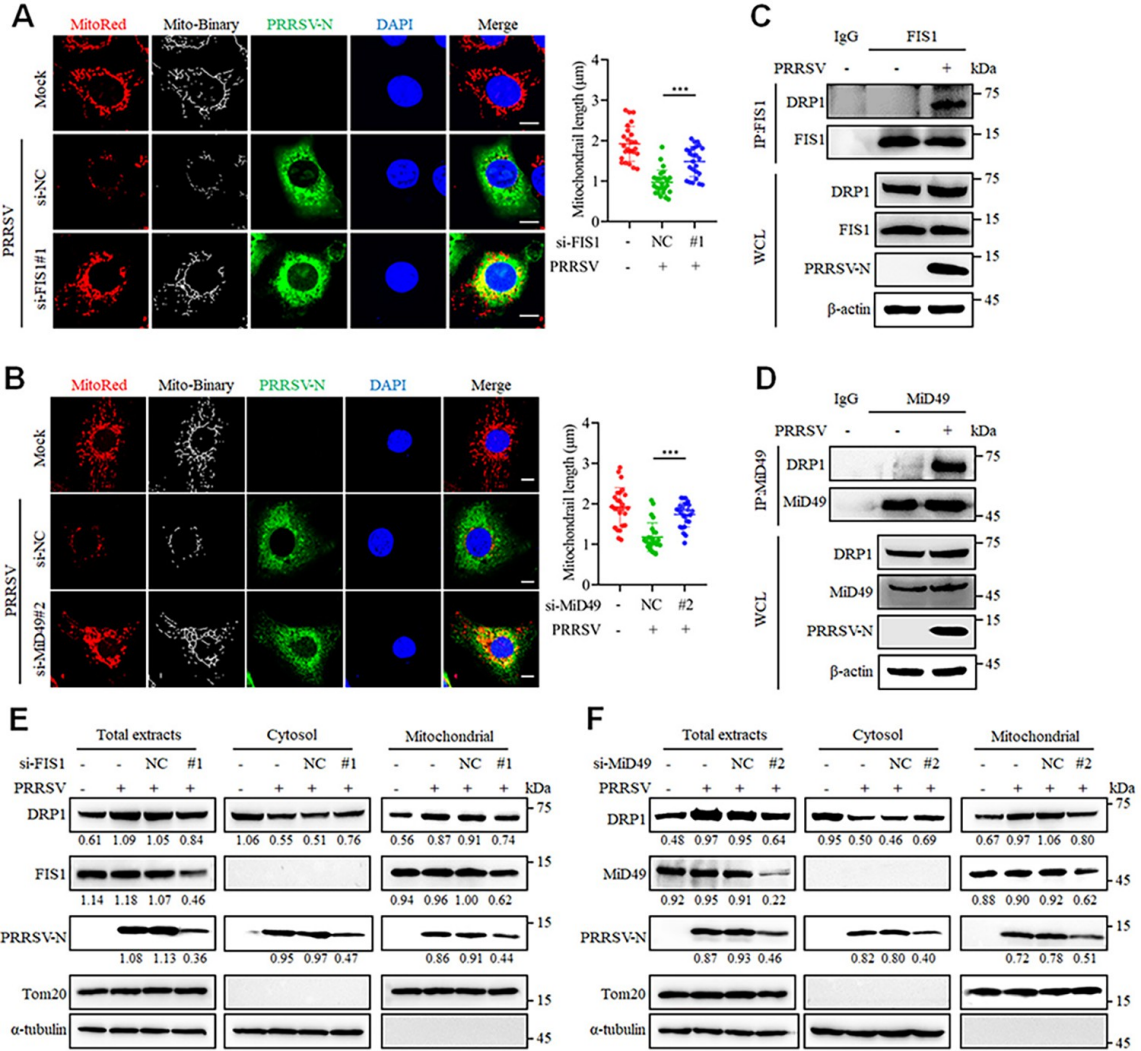
FIS1 and MiD49 as the adaptors of DRP1 mediates PRRSV-induced mitochondrial fission. (A-B) Marc-145 cells were transfected with control siRNA or siRNA targeting FIS1 (A) or MiD49 (B) for 24 h, and then were either mock-infected or infected with PRRSV (0.1 MOI) for an additional 24 h. The cells were stained with MitoTracker Red, PRRSV-N antibody, and DAPI, followed by confocal microscopy. Quantitative analysis of mitochondrial length is presented for 25 cells per group (right panel). (C-D) Marc-145 cells were mock-infected or infected with PRRSV (MOI = 0.1) for 24 h. The cells were lysed and precipitated using anti-FIS1 or anti-MiD49 antibodies. The whole-cell lysates (WCL) and immunoprecipitated proteins were analyzed using specific antibodies. Anti-Rabbit immunoglobulin G (IgG) antibody was used as a negative control. (E and F) After 24 hours of siRNA FIS1 (E) and MiD49 (F) transfection, the cells were either mock-infected or infected with PRRSV (0.1 MOI) for an additional 24 hours, and then harvested for cytoplasmic and mitochondrial fractionation. Then samples were subjected to western blot analysis. The levels of proteins were normalized to Tom20 or α-tubulin. Data are expressed as means ± SD, n = 25 in A and B. *p<0.05; **p < 0.01; ***p < 0.001. The data are representative of results from three independent experiments.

The movement of DRP1 to the mitochondria is orchestrated by the engagement of specific adaptors, including FIS1, MFF, and two mitochondrial dynamic proteins (MiD49 and MiD51, [33]). To investigate whether these four adaptors facilitate mitochondrial fission during PRRSV infection, specific siRNAs were designed (S5 Fig). Confocal microscopy results demonstrated that the knockdown of FIS1 and MiD49 significantly impeded PRRSV-induced mitochondrial fission (Fig 7A and 7B), while the knockdown of MFF and MiD51 did not exhibit this effect (S6A and S6B Fig). Consistently, robust interactions between DRP1 and FIS1, as well as DRP1 and MiD49, were observed in PRRSV-infected cells (Fig 7C and 7D), whereas no interaction were detected with MFF and MiD51 (S6C and S6D Fig). Furthermore, we found that knockdown of FIS1 and MiD49, but not MFF and MiD51, reversed the accumulation of DRP1 on the mitochondria during PRRSV infection and inhibited its replication (Figs 7E–7F and S6E–S6J). These results suggest that FIS1 and MiD49 contribute to mitochondrial fission, and viral replication after PRRSV infection.

### Mitochondrial Ca^2+^ influx induces mitophagy mediated by the PINK1-Parkin pathway

**Fig 8:**
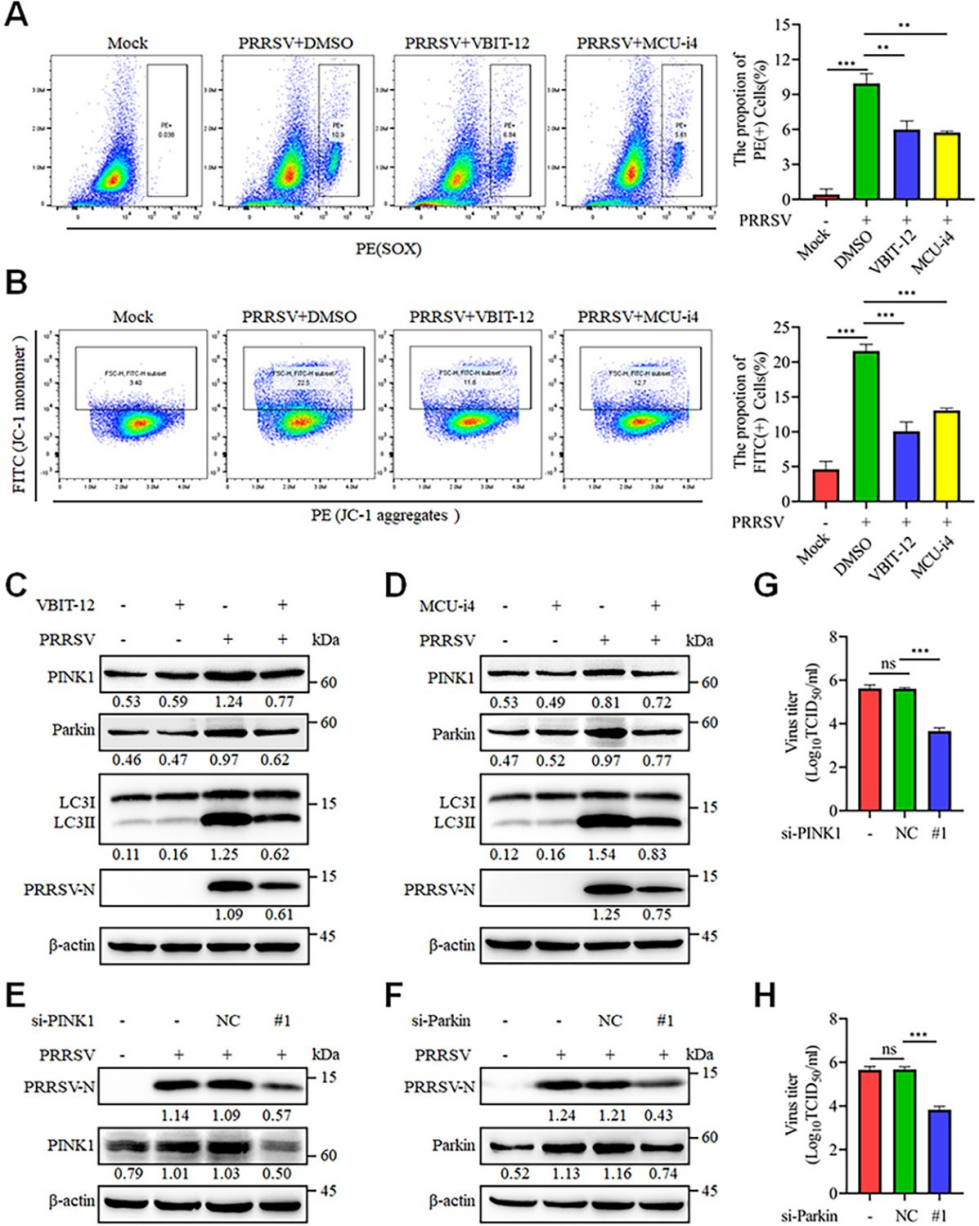
Mitochondrial Ca^2+^ triggers PRRSV-induced mitophagy via the PINK1-Parkin pathway. (A-D) Marc-145 cells were mock-infected or PRRSV-infected (MOI = 0.1) for 24 h with VBIT-12 (10 μM) or MCU-i4 (10 μM). (A) SOX was detected by flow cytometry of MitoSOX Red-stained cells. Statistical analysis of the positive cell population is presented (right panel). (B) The mitochondrial membrane potential was detected by flow cytometry of JC-1-stained cells. Statistical analysis of the positive cell population is presented (right panel). (C-D) Cell lysates were harvested for western blot analysis with antibodies against PINK1, Parkin, LC3, PRRSV-N and β-actin. (E-H) After 24 hours of siRNA transfection, the cells were either mock-infected or infected with PRRSV (0.1 MOI) for an additional 24 hours. (E-F) The cell lysates were harvested for western blot analysis with antibodies against PRRSV-N, PINK1 (for E), Parkin (for F) and β-actin. (G-H) Determination of the TCID_50_ of PRRSV in cell supernatants. Data are expressed as means ± SD, n = 3 in A, B, G and H. *p<0.05; **p < 0.01; ***p < 0.001. The levels of proteins were normalized to β-actin. The data are representative of results from three independent experiments.

The PRRSV-induced mitochondrial fission and decreased abundance suggest impaired mitochondrial health. To further assess this, MitoTracker Red was used, as its fluorescence intensity correlates with mitochondrial membrane potential. As shown in the figures above, PRRSV infection significantly reduced fluorescence intensity, indicating a decrease in mitochondrial membrane potential. Typically, changes in mitochondrial membrane potential coincide with the induction of ROS, leading to mitochondrial dysfunction [34]. To investigate whether PRRSV infection promotes changes in ROS levels and mitochondrial membrane potential, we stained infected cells with MitoSOX and JC-1. Flow cytometry results indicated that PRRSV infection promoted the generation of superoxide (SOX, a type of ROS) and disrupted mitochondrial membrane potential (indicated by JC-1 staining) (S7A and S7B Fig). As excessive Ca^2+^ is a contributing factor to mitochondrial fission, we conducted further investigations into the connection between Ca^2+^ and mitochondrial dysfunction. As anticipated, the Ca^2+^ mitochondrial entry channel inhibitors VBIT-12 and MCU-i4 significantly inhibited PRRSV-induced SOX production and partially preserved the impaired mitochondrial membrane potential (Fig 8A and 8B). These findings verify that the excessive mitochondrial Ca^2+^ plays a role in mitochondrial dysfunction during PRRSV infection.

To preserve homeostatic balance, damaged or dysfunctional mitochondria are selectively degraded through a process known as mitophagy [35]. To examine whether PRRSV infection triggers mitophagy, Marc-145 cells were transfected with the autophagy marker GFP-LC3 and then mock-infected or infected with PRRSV. Subsequently, mitochondria were labeled with the mitochondrial marker Translocase of Outer Mitochondrial Membrane 20 (Tom20) and visualized by confocal microscopy analysis. Upon PRRSV infection, the cells displayed a distinct punctate distribution of GFP-LC3, with significant colocalization with Tom20, suggesting that PRRSV infection induces mitophagy (S7C Fig).

For further verification, we examined the classical ubiquitin-dependent and non-ubiquitin-dependent autophagy receptors for mitophagy, including PINK1, Parkin, NIX, OPTN, NDP52 and p62 [35]. Only PINK1 and Parkin were upregulated by PRRSV infection at the mRNA and protein levels, indicating that the PINK1/Parkin pathway is involved in PRRSV-induced mitophagy (S7D–S7F Fig). Notably, Tom20 levels remained unchanged at 24 hours post-PRRSV infection but showed significant degradation at 36 hours, suggesting the involvement of distinct forms of autophagy during PRRSV infection (S7G Fig). Furthermore, inhibition of Ca^2+^ influx into mitochondria by treatment with VBIT-12 and MCU-i4 significantly impeded the PRRSV-induced expression of PINK1/Parkin and conversion of LC3II (Fig 8C and 8D). Moreover, siRNAs targeting PINK1 and Parkin had no impact on cell viability (S8 Fig) but resulted in a significant reduction in PRRSV replication, as evidenced by decreased PRRSV-N expression and viral titers (Fig 8E–8H). Collectively, these results suggest that the excessive mitochondrial Ca^2+^ during PRRSV infection induces mitochondrial dysfunction and mitophagy, thereby promoting its replication.

### Mitochondrial fission and mitophagy, both mediated by Ca^2+^, promote glycolysis

Being the primary site for ATP production, mitochondria undergo changes in cellular energy metabolism in response to damage [36]. Therefore, we investigated alterations in oxidative phosphorylation (OXPHOS) and glycolytic processes in cells infected with PRRSV. Compared to uninfected cells, PRRSV-infected cells exhibited a significant decrease in the Oxygen Consumption Rate (OCR), ATP production and basal respiratory capacity (S9A–S9C Fig); and an increased Extracellular Acidification Rate (ECAR) (S9D–S9F Fig), indicating an enhancement of both basal glycolysis and compensatory glycolytic capacity.

**Fig 9:**
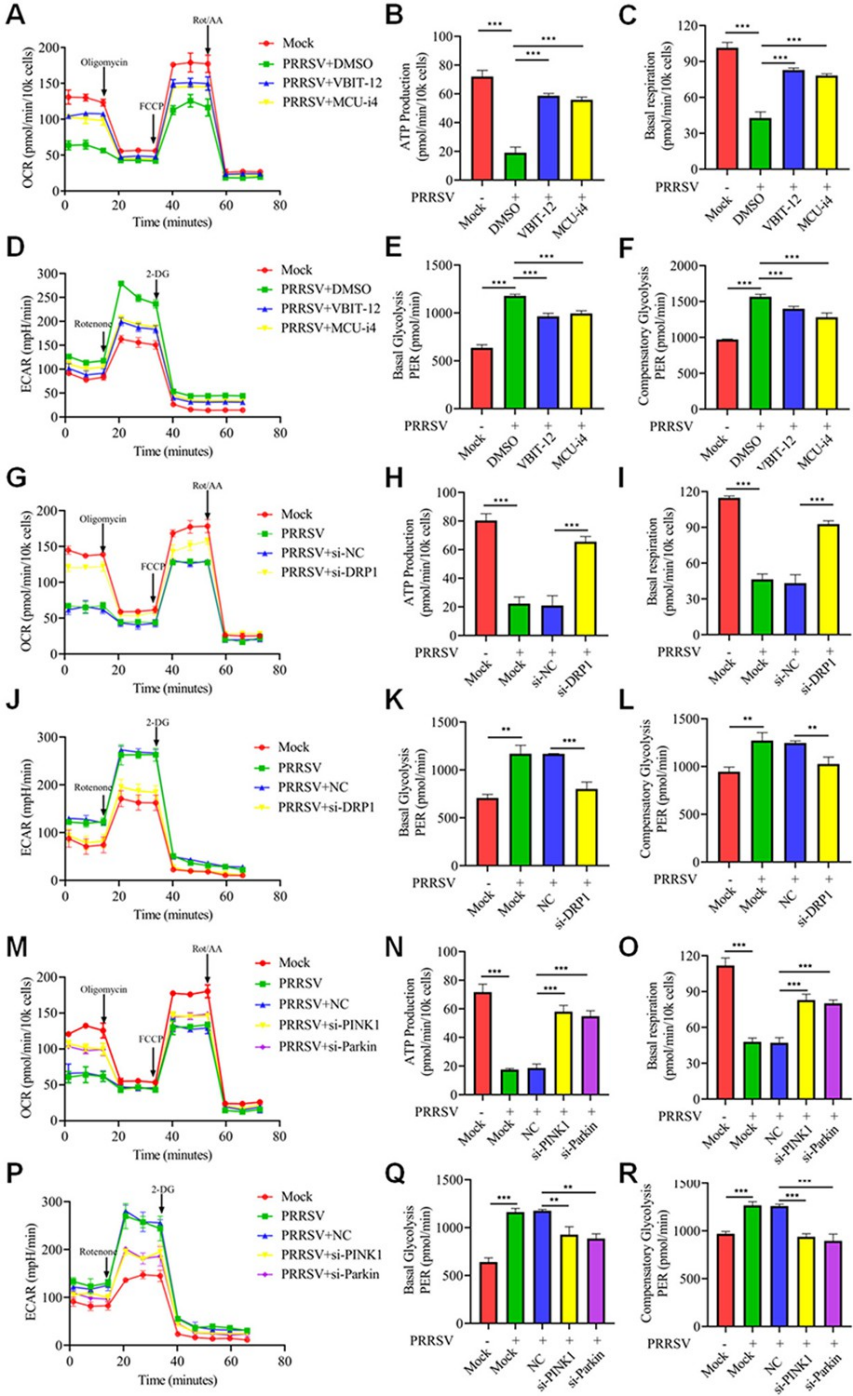
Mitochondrial Ca^2+^, mitochondrial fission and mitophagy promote glycolysis. (A-F) Marc-145 cells were mock-infected or PRRSV-infected (MOI = 0.1) for 24 h without or with the addition of VBIT-12 (10 μM) or MCU-i4 (10 μM). OCR and ECAR assays were conducted, and statistical analysis was performed to assess cellular ATP production, basal respiration, basal glycolysis and compensatory glycolytic capacity. (G-R) After 24 hours of siRNA transfection, cells were either infected with PRRSV (0.1 MOI) or left uninfected for an additional 24 hours. OCR and ECAR measurements were conducted, and statistical analysis of the cellular ATP production capacity and basal respiration capacity or glycolytic capacity was conducted. Data are expressed as means ± SD (n = 3). *p<0.05; **p < 0.01; ***p < 0.001. The data are representative of results from three independent experiments.

Mitochondrial Ca^2+^ acts as a cofactor in ATP synthesis for cellular energy production [37,38]. Therefore, we investigated the impact of mitochondrial Ca^2+^ on OXPHOS and glycolytic processes induced by PRRSV infection. The results showed that inhibition of Ca^2+^ mitochondrial influx using VBIT-12 and MCU-i4 ablated the effect of PRRSV infection in inhibiting OXPHOS and promoting glycolysis processes (Fig 9A–9F). To further explore the relationship between PRRSV-induced mitochondrial fission and cellular glucose metabolism, we employed siRNA targeting DRP1. DRP1 knockdown significantly ameliorated basal respiratory capacity and ATP production in PRRSV-infected cells, thus restoring the cellular OXPHOS (Fig 9G–9I). Moreover, siRNA targeting DRP1 mediated a corresponding inhibition of the glycolytic process in PRRSV-infected cells (Fig 9J–9L). Similar results were obtained upon Mdivi-1 treatment (S9G–S9L Fig). Consistently, inhibition of mitophagy by knocking down PINK1 or Parkin also promoted OXPHOS and suppressed glycolysis (Fig 9M–9R). These results suggest that PRRSV-induced mitochondria fission and mitophagy, mediated by mitochondrial Ca^2+^, are accompanied by disruption of cellular OXPHOS and increased glycolytic processes.

**Fig 10:**
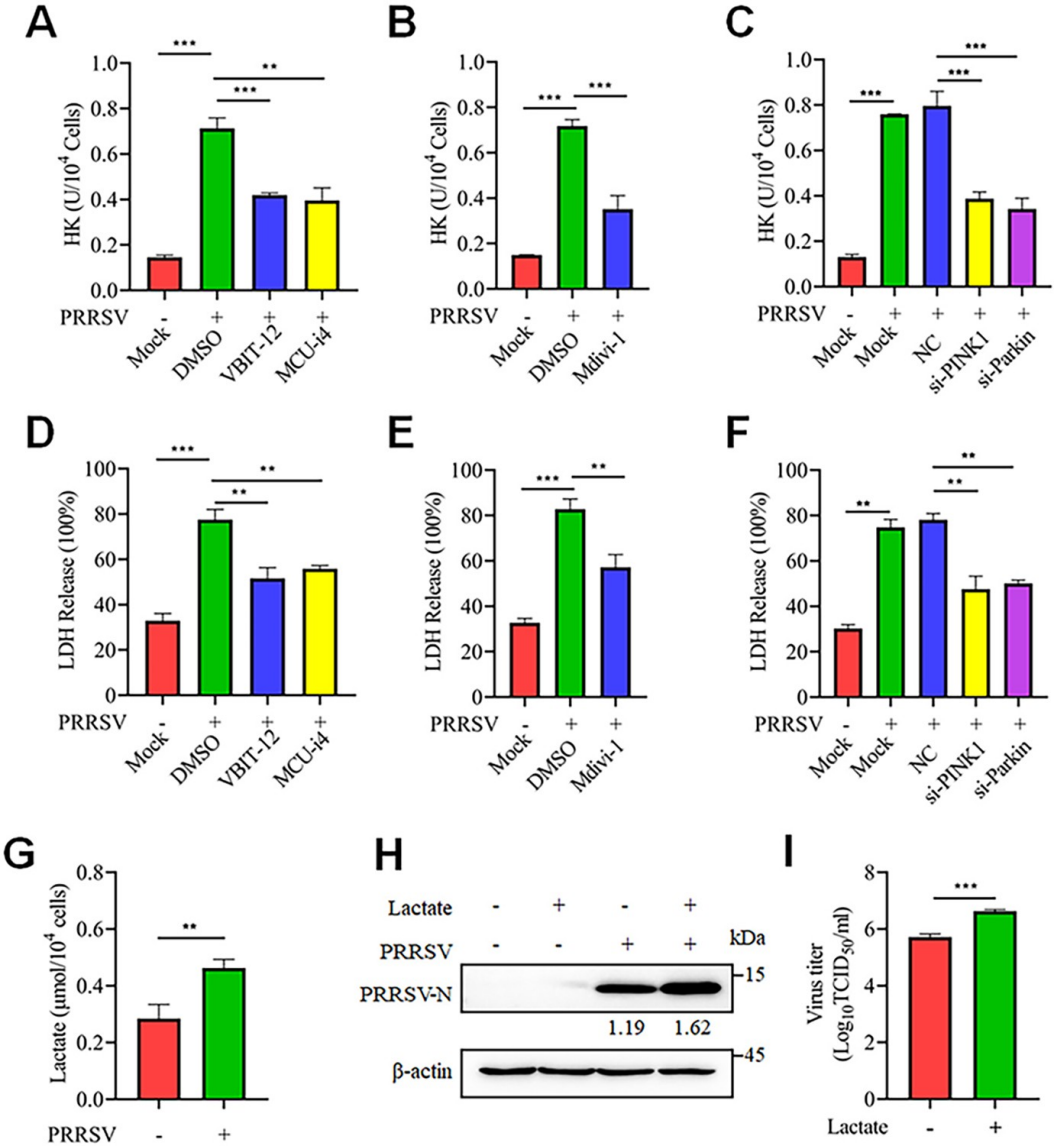
PRRSV exploits glycolysis to promote its own replication. (A-F) In Marc-145 cells, after drug treatment (VBIT-12, MCU-i4 or Mdivi-1) or siRNA transfection for 24 hours, followed by simulated or PRRSV infection for an additional 24 hours, the levels of HK or LDH were measured. (G) Marc-145 cells were infected with PRRSV (MOI = 0.1) for 24 hours, and then the intracellular lactate levels were measured. (H and I) Marc-145 cells were treated with lactate (20 mM) and then either mock-infected or infected with PRRSV (MOI = 0.1) for 24 hours. (H) The cell lysates were harvested for western blot analysis with antibodies against PRRSV-N and β-actin. The level of PRRSV-N was normalized to β-actin. (I) Determination of the TCID_50_ of PRRSV in the cell supernatant. Data are expressed as means ± SD (n = 3). *p<0.05; **p < 0.01; ***p < 0.001. The data are representative of results from three independent experiments.

Next, we investigated how PRRSV utilizes Ca^2+^ and mitophagy to influence glycolysis. Our findings indicated that PRRSV significantly upregulated the activity of two key kinases in glycolysis: hexokinase (HK) and lactate dehydrogenase (LDH) (S9M and S9N Fig). The enhancement was effectively eliminated by using VBIT-12 and MCU-i4 to reduce Ca^2+^ influx into the mitochondria (Fig 10A and 10D), as well as by inhibiting DRP1 with Mdivi-1 and knocking down PINK1 and Parkin using siRNAs (Fig 10B, 10C, 10E and 10F).

LDH catalyzes the conversion of pyruvate to lactate, an important metabolite that plays a crucial role in both physiological and pathological processes [39,40]. Considering the recently revealed role of lactate in the infection mechanisms of various viruses [41,42], we investigated whether lactate bridges glycolysis and PRRSV replication. Our results showed that lactate levels were significantly increased in PRRSV-infected cells (Fig 10G). Furthermore, the exogenous addition of lactate notably promoted PRRSV replication (Fig 10H and 10I). Together, these results indicate that PRRSV upregulates the activity of HK and LDH through Ca^2+^-induced mitochondrial fission and mitophagy, thereby enhancing cellular glycolysis and increasing lactate production, which in turn promotes its own replication.

### PRRSV infection triggers mitochondrial fission and mitophagy mediated by mitochondrial Ca^2+^ to promote glycolysis in primary cells

**Fig 11:**
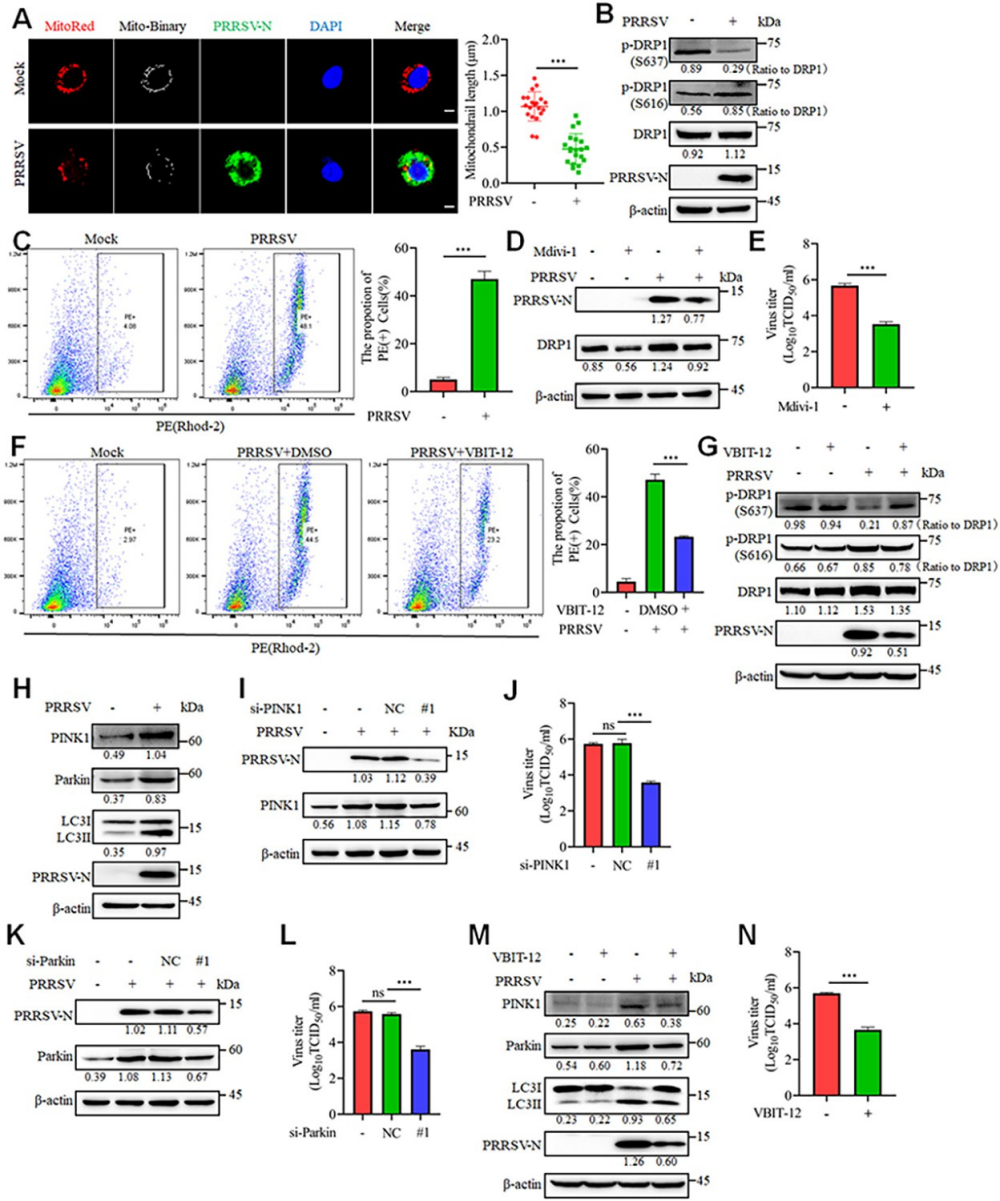
PRRSV infection triggers mitochondrial fission and mitophagy mediated by mitochondrial Ca^2+^ in primary cells. (A-C) PAMs were mock-infected or PRRSV-infected (MOI = 0.1) for 18 h. (A) The cells were stained with MitoTracker Red, PRRSV-N antibody and DAPI, followed by confocal microscopy. Quantitative analysis of mitochondrial length is presented on the right. The lengths of mitochondria from 20 cells were quantified for each group. (B) Cell lysates were harvested for western blot analysis. (C) Detection of mitochondrial Ca^2+^ using Rhod-2 staining by flow cytometry. (D and E) PAMs were exposed to PRRSV (MOI = 0.1) for 18 hours, either with or without Mdivi-1 (2.5 μM) treatment. (D) Western blot analysis was performed on the cell lysates by using antibodies against DRP1, PRRSV-N and β-actin. (E) Assessment of the TCID_50_ of PRRSV in the cell supernatant. (F and G) PAMs were either treated or not treated with VBIT-12 (5 μM), then mock-infected or infected with PRRSV (MOI = 0.1) for 18 hours. (F) Ca^2+^ levels were assessed using flow cytometry with Rhod-2 staining. (G) The cell lysates underwent western blot analysis using antibodies specific to DRP1 Ser637, DRP1 Ser616, DRP1, PRRSV-N and β-actin. (H) PAMs were mock-infected or infected with PRRSV (MOI = 0.1) for 18 hours. Collect cell lysates and perform western blot analysis using antibodies against PINK1, Parkin, LC3, PRRSV-N, and β-actin. (I-L) PAMs cells were transfected with siRNA targeting PINK1 (I and J) or Parkin (K and L) for 24 hours, followed by 18 hours of mock or PRRSV infection (MOI = 0.1). (I and K) Cell lysates were harvested for western blot analysis with antibodies against PINK1 or Parkin, PRRSV-N and β-actin. (J and L) Determination of the TCID_50_ of PRRSV in the cell supernatant. (M and N) PAMs were either treated or not treated with VBIT-12 (5 μM), then mock-infected or infected with PRRSV (MOI = 0.1) for 18 hours. (M) Cell lysates were harvested for western blot analysis with antibodies against PINK1, Parkin, LC3, PRRSV-N, and β-actin. (N) Determination of the TCID_50_ of PRRSV in the cell supernatant. Data are expressed as means ± SD, n = 20 in A or n = 3 in C, E, F, J, L, N. *p<0.05; **p < 0.01; ***p < 0.001. The levels of phosphorylated DRP1 were normalized to the total DRP1 protein, while the levels of other proteins were normalized to β-actin. The data are representative of results from three independent experiments.

**Fig 12:**
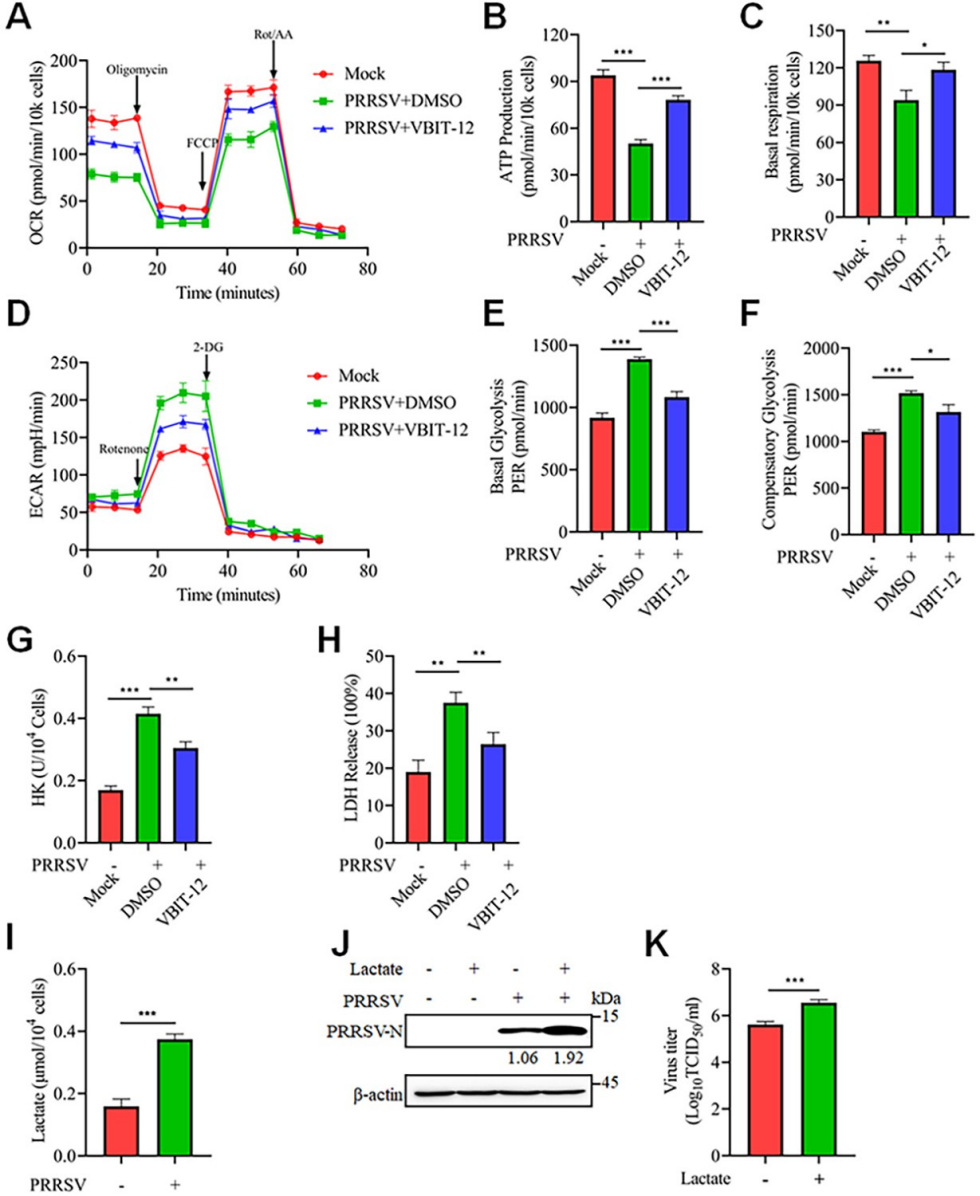
PRRSV promotes its replication in primary cells by stimulating glycolysis. (A-F) After treating PAMs with VBIT-12, cells were either mock-infected or infected with PRRSV (MOI = 0.1) for 18 hours. Subsequently, OCR and ECAR measurements were performed to assess cellular ATP production, basal respiration, basal glycolysis, and compensatory glycolysis capacity. (G and H) PAMs were pretreated with VBIT-12 (5 μM), followed by mock or PRRSV infection (MOI = 0.1) for 18 hours. Then, the levels of HK (G) and LDH (H) in the cells were measured. (I) After infecting PAMs with PRRSV at an MOI of 0.1 for 18 hours, lactate levels in the cells were measured using the CheKine Micro Lactate Assay Kit. (J and K) PAMs were pretreated with 10 mM lactate for 2 hours, followed by mock or PRRSV infection (MOI = 0.1) for 18 hours. (J) Cell lysates were collected for western blot analysis with antibodies against PRRSV-N and β-actin. The level of PRRSV-N was normalized to β-actin. (K) Quantification of the TCID_50_ of PRRSV in cell supernatants. Data are expressed as means ± SD (n = 3). *p<0.05; **p < 0.01; ***p < 0.001. The data are representative of results from three independent experiments.

PRRSV exhibits a preference for infecting cells of the monocyte/macrophage lineage *in vivo*, with porcine alveolar macrophages (PAMs) as its primary target [43]. Therefore, we investigated whether PRRSV infection of PAMs had similar effects on mitochondrial fission. PRRSV infection resulted in a reduction in mitochondrial length in PAMs, indicative of sustained mitochondrial fission (Fig 11A). Mitochondrial fission was also induced by PRRSV through decreasing DRP1 phosphorylation at Ser637 and increasing DRP1 phosphorylation at Ser616 (Fig 11B). Additionally, PRRSV infection markedly increased the mitochondrial Ca^2+^ levels in PAMs (Fig 11C). Next, we explored the relationship between mitochondrial fission, Ca^2+^ levels, and PRRSV replication. By using the DRP1 inhibitor Mdivi-1, PRRSV replication was significantly inhibited (Fig 11D and 11E). Moreover, VBIT-12, a specific inhibitor of VDAC, significantly suppressed the PRRSV-induced increase in mitochondrial Ca^2+^ levels (Fig 11F). Consistently, the administration of VBIT-12 effectively inhibited the reduction of DRP1 Ser637 phosphorylation and the elevation of DRP1 Ser616 phosphorylation induced by PRRSV infection, and concurrently suppressed PRRSV replication (Fig 11G). In addition, we investigated whether PRRSV was also capable of inducing mitophagy in PAMs. As anticipated, PRRSV infection significantly upregulated the expression levels of PINK1 and Parkin, and promoted the conversion of LC3I to LC3II, suggesting that PRRSV infection induces mitophagy in PAMs (Fig 11H). Importantly, silencing PINK1 or Parkin significantly inhibited PRRSV replication, highlighting the crucial role of mitophagy in PRRSV replication (Fig 11I–11L). Moreover, VBIT-12 significantly inhibited PRRSV replication by reducing PINK1 and Parkin levels (Fig 11M and 11N). Consistent with observations in Marc-145 cells, PRRSV infection significantly inhibited OXPHOS in PAMs while promoting glycolysis (S10A–S10F Fig). Surprisingly, VBIT-12 treatment significantly improved the basal respiratory capacity and ATP production in PRRSV-infected cells, restoring cellular oxidative phosphorylation levels (Fig 12A–12C). Consistently, VBIT-12 inhibited the glycolytic process in PRRSV-infected cells (Fig 12D–12F). Crucially, we found that PRRSV infection promoted glycolysis by increasing the production of HK and LDH in PAMs, which in turn led to elevated lactate production (Fig 12G–12I). Additionally, the addition of lactate promoted PRRSV replication in PAMs (Fig 12J and 12K). In summary, these results suggest that PRRSV promotes its own replication by increasing mitochondrial Ca^2+^ levels, which induces mitochondrial fission and mitophagy to manipulate the glycolytic process.

## Discussion

The ER and mitochondria, functioning as crucial cellular Ca^2+^ storage organelles and regulators of cellular Ca^2+^ homeostasis, play pivotal roles in viral infections. In our previous study, we found that PRRSV infection induces ER stress and increases cytoplasmic Ca^2+^ levels, thereby activating autophagy through the CaMKII-AMPK-mTOR-LC3II pathway to support its replication [23]. In this study, we further demonstrated that PRRSV infection induces the transfer of Ca^2+^ from the ER to the mitochondria, leading to mitochondrial fission, mitophagy, and enhanced glycolysis, ultimately promoting viral replication. Remarkably, our observations revealed that PRRSV infection leads to an augmentation of MAMs, providing a mechanism for Ca^2+^ exchange between the two organelles [27,28]. Notably, Zhang et al. demonstrated that PRRSV GP5 is involved in enhancing MAMs and mitochondrial fission [24]. Based on these findings, we hypothesize that GP5 may play a role in PRRSV-induced mitophagy and glycolysis, which warrants further investigation. Our findings are consistent with those of other studies demonstrating that viruses disrupt intracellular Ca^2+^ homeostasis through adaptation or inhibition of Ca^2+^ signaling pathways, creating favorable cellular conditions for the replication of progeny viruses [44,45].

Our results suggest that PRRSV-activated DRP1 regulates mitochondrial fission, as confirmed using the specific inhibitor Mdivi-1 and DRP1 siRNA. DRP1 has also been shown to mediate the infection processes of other viruses. For example, Coxsackie virus B induces mitochondrial fission via the DRP1 pathway, facilitated by TRPV1, a transient receptor potential channel, ultimately facilitating a pro-viral effect [46], while HCV triggers DRP1 phosphorylation, leading to its translocation to mitochondria and subsequent fission activation [16]. In contrast, dengue virus, a member of the Flaviviridae family, inhibits DRP1-mediated mitochondrial fission to support its replication [47], and influenza virus alters ER-mitochondria interactions in infected cells, requiring the repositioning of DRP1 to the cytosol [17]. In our current investigation, we discovered that PRRSV infection induces DRP1 S637 dephosphorylation and S616 phosphorylation via the CaMKKβ-AMPK axis. Inhibition of CaMKKβ and AMPK by inhibitors or siRNAs suppresses PRRSV replication. However, previous research by Fang et al. observed that AMPK activation can inhibit PRRSV replication [48]. These findings suggest that AMPK plays a dual role in PRRSV infection at different replication stages, which warrants further investigation. Furthermore, we discovered that activation of the CaMKKβ-AMPK signaling pathway is facilitated by elevated mitochondrial Ca^2+^ released from ER via the IP3R-VDAC1-MCU channels. Our results indicate that DRP1 is subsequently translocated to the mitochondria, where it interacts with FIS1 and MiD49 to initiate fission and promote PRRSV replication. DRP1 has also been identified to undergo posttranslational modifications other than phosphorylation, such as SUMOylation and ubiquitination [49,50], which may contribute to the upregulated expression of DRP1 in response to PRRSV infection.

Mitophagy, a protective cellular mechanism, maintains mitochondrial homeostasis by removing excess or dysfunctional mitochondria, with PINK1 and Parkin playing key roles in this process [10]. We demonstrated that PRRSV-induced Ca^2+^ influx promotes mitophagy via the PINK1/Parkin pathway. While only a few viruses have been reported to inhibit mitophagy, many have evolved to hijack it to facilitate their replication. For instance, hepatitis B virus induces mitophagy by upregulating PINK1 and Parkin expression, thus inhibiting apoptosis to support viral persistence [51]. Similarly, HCV-induced mitochondrial fission is followed by Parkin-mediated mitophagy [16,51]. Interestingly, during SARS-CoV-2 infection, USP30 acts as a mitochondrial deubiquitinase to suppress PINK1-mediated mitophagy; however, SARS-CoV-2 also directly stimulates PINK1 expression, potentially influencing mitophagy progression [52]. The relationship between PRRSV infection and autophagy is complex and multifaceted. Chen et al. first discovered that PRRSV induces autophagy and forms complete autolysosomes, which are essential for viral replication [53], and Liu et al. later confirmed this finding [54]. However, Sun et al. showed that the PRRSV Nsp2 protein enhances viral replication by preventing the fusion of autophagosomes with lysosomes [55]. Similarly, studies by Zhou and Li et al. demonstrated that the PRRSV Nsp5 and GP5 proteins sustain viral infection by inhibiting the progression of autophagic flux [56,57]. In our study, we found that PRRSV infection activates the PINK1/Parkin-mediated mitophagy pathway 24 hours post-infection, but it does not result in the degradation of Tom20. Interestingly, silencing PINK1 and Parkin significantly reduced viral replication, highlighting the importance of mitophagy activation for PRRSV infection, even though the autophagic process was incomplete. At 36 hours post-infection, we observed a marked decrease in Tom20 levels, suggesting complete autophagy had occurred. In summary, PRRSV infection involves a dynamic autophagic process. While previous research has shown that other viruses exploit autophagy at different stages to maximize replication, the specific mechanisms of this strategy in PRRSV infection require further investigation.

As a secondary intracellular messenger, Ca^2+^ participates in a variety of physiological and biochemical processes, such as cell growth and proliferation, energy metabolism, and immune response [37,58,59]. In this study, we demonstrated a role for PRRSV-induced Ca^2+^ in the regulation of glycolysis. Glycolysis is a common metabolic program during viral infection. For instance, Dengue virus induces the expression of GLUT1 and HK2 to enhance glucose uptake and downstream glycolytic processes, promoting viral replication [60]. Similarly, Influenza A virus utilizes HK2 and pyruvate kinase M2 to boost glycolysis and further support viral replication [61]. Additionally, increased glycolysis via the Akt signaling pathway has been observed in murine Norovirus-infected macrophages [62]. In the case of PRRSV, we demonstrated that infection heightens cellular glucose uptake and promotes the glycolytic process by upregulating HK and LDH activity, enhancing lactate production, and thereby facilitating viral replication. Notably, further research is needed to determine which PRRSV protein is responsible for upregulating both enzyme activities. Consistently, lactate has been reported to promote PRRSV replication through interferon pathway by targeting heat shock 70 kDa protein 6 (HSPA6) and mitochondrial antiviral-signaling protein (MAVS) [63,64]. The beyond roles of lactate in PRRSV infection remain to be elucidated.

**Fig 13:**
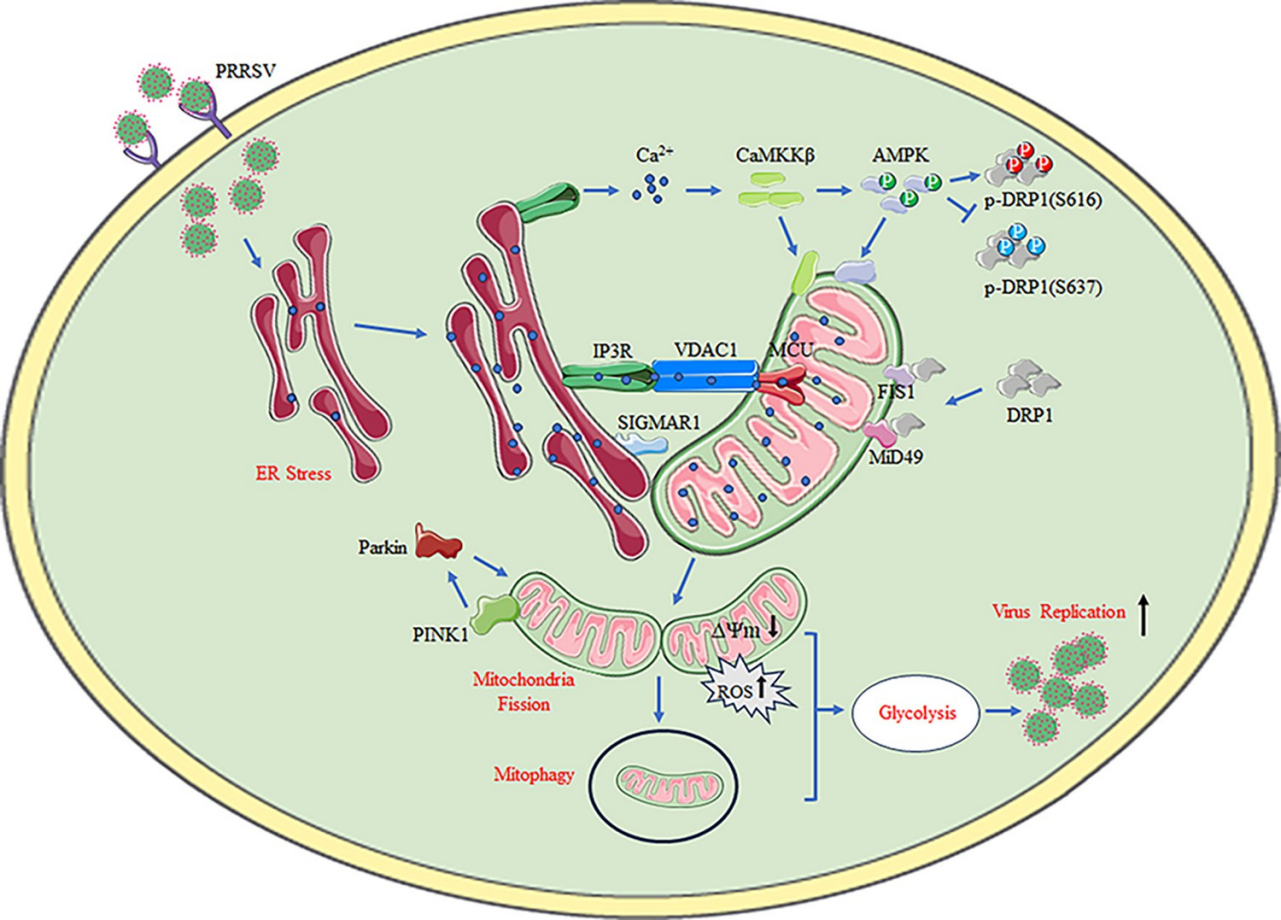
Model of calcium-mediated mitochondrial fission, mitophagy and glycolysis to facilitate PRRSV replication. Upon cellular invasion, PRRSV induces ER stress and promotes the formation of MAMs via SIGMAR1, facilitating Ca^2+^ influx into the mitochondria through the IP3R-VDAC1-MCU channel. Excessive Ca^2+^ induces DRP1 S637 dephosphorylation and S616 phosphorylation via CaMKKβ-AMPK, leading to sustained mitochondrial fission, mitochondrial dysfunction, and activation of the PINK1-Parkin pathway for mitophagy clearance of damaged mitochondria. Concurrently, mitochondrial damage and mitophagy impair oxidative phosphorylation and enhance glycolysis, ultimately promoting PRRSV replication. Fig 13 was modified from Servier Medical Art (https://smart.servier.com/), licensed under a Creative Commons Attribution 4.0 International (CC BY) license (https://creativecommons.org/licenses/by/4.0/).

In summary, we described a mechanism in which Ca^2+^-mediated mitochondrial fission and mitophagy induce glycolysis to facilitate PRRSV replication (Fig 13). Upon cellular invasion, PRRSV induces ER stress and promotes the formation of MAMs via SIGMAR1, enabling Ca^2+^ influx into mitochondria via the IP3R-VDAC1-MCU channel. Excessive Ca^2+^ induces DRP1 phosphorylation changes through the CaMKKβ-AMPK pathway, leading to sustained mitochondrial fission, mitochondrial dysfunction, and activation of the PINK1-Parkin pathway for the mitophagic clearance of damaged mitochondria. Concurrently, mitochondrial fission and mitophagy impair oxidative phosphorylation and enhance glycolysis, ultimately promoting PRRSV replication. This study bridges the connection between mitochondrial Ca^2+^, mitochondrial dynamics, and metabolism during viral infection, providing new insights into viral control. On the basis of these findings, pharmacological blockade of Ca^2+^ channels may provide an effective strategy for controlling PRRSV and other viral infections.

## Materials and methods

### Ethics statement

The animal experiments conformed to the rules of National Guidelines for Housing and Care of Laboratory Animals (China) and were performed after obtaining the approval of the Institutional Animal Care and Ethics Committee of Nanjing Agricultural University (permit no. IACECNAU20191002).

### Cells and viruses

Marc-145 cells, a permissive cell line for PRRSV infection *in vitro*, were obtained from the American Type Culture Collection and cultured in Dulbecco’s modified Eagle’s medium (DMEM; Gibco, 11,995,065) supplemented with 10% fetal bovine serum (FBS; Gibco, 10,099,141), 100 U/ml penicillin, and 100 μg/ml streptomycin (Sigma Aldrich, P4333). PAMs were isolated from specific-pathogen-free 4-week-old piglets using established protocols [65] and maintained in RPMI 1640 medium (Gibco, 11,875,093), supplemented with 10% FBS, 100 U/ml penicillin, and 100 μg/ml streptomycin. All cells were cultured in a humidified 5% CO_2_ incubator at 37°C. The highly pathogenic PRRSV-2 isolate BB0907 [66] (GenBank accession no. HQ315835.1), referred to as “PRRSV” in this investigation, was amplified and titrated in Marc-145 cells.

### Antibodies and chemical reagents

The antibodies used in this study were as follows: anti-PRRSV-N protein (produced in our laboratory), anti-β-actin (Proteintech, 66009-1-Ig), anti-DRP1 (Cell Signaling Technology, 8570), anti-p-DRP1 S616 (Cell Signaling Technology, 3455), anti-p-DRP1 S637 (Cell Signaling Technology, 4867), anti-AMPKα (Cell Signaling Technology, 2532), anti-p-AMPKα Thr172 (Cell Signaling Technology, 2535), anti-CaMKKβ (Proteintech, 11549-1-AP), anti-Tom20 (Proteintech, 11802-1-AP), anti-α-tubulin (Proteintech, 11224-1-AP), anti-FIS1 (Proteintech, 10956-1-AP), anti-MFF (Proteintech, 17090-1-AP), anti-MiD49 (Proteintech, 28718-1-AP), anti-MiD51 (Proteintech, 20164-1-AP), anti-LC3 (Proteintech, 14600-1-AP), anti-PINK1 (Proteintech, 23274-1-AP), anti-Parkin (Proteintech, 14060-1-AP), anti-PERK (Cell Signaling Technology, 3192), anti-p-PERK (Thr980) (Cell Signaling Technology, 3179), anti-eIF2α (Cell Signaling Technology, 9722), anti-p-eIF2α (Ser51) (Cell Signaling Technology, 9721), anti-SIGMAR1 (Proteintech, 15168-1-AP), anti-FUNDC1 (Proteintech, 28519-1-AP), anti-Calcineurin (CaN) (Proteintech, 13422-1-AP), anti-Calnexin (Proteintech, 10427-2-AP), anti-NIX (Proteintech, 68118-1-Ig), anti-NDP52 (Proteintech, 12229-1-AP), anti-OPTN (Proteintech, 10837-1-AP), anti-p62 (Proteintech,18420-1-AP), anti-GRP78 (Cell Signaling Technology, 3177), Mouse monoclonal [SB62a] Anti-Rabbit IgG light chain (HRP) (Abcam, ab99697), Goat Anti-Rabbit IgG H&L (HRP) (Abcam, ab6721), Goat Anti-Mouse IgG H&L (HRP) (Abcam, ab205719), CoraLite488-conjugated Goat Anti-Rabbit IgG (H+L) (Proteintech, SA00013-2), CoraLite488-conjugated Goat Anti-Mouse IgG (H+L) (Proteintech, SA00013-1), CoraLite594-conjugated Goat Anti-Rabbit IgG (H+L) (Proteintech, SA00013-4), and Goat Anti-Mouse IgG H&L (Alexa Fluor 647) (Abcam, ab150115). The following chemicals were purchased from Selleck: Mdivi-1 (S7162), 2-APB (S6657), VBIT-12 (S8936), MCU-i4 (S9842), and FK506 (S5003).

### Transmission electron microscopy

Marc-145 cells were subjected to mock infection or infection with PRRSV at an MOI of 0.1 for 24 hours. Subsequently, the cells were collected and fixed in 2.5% glutaraldehyde at 4°C for 12 hours, followed by post-fixation in 1% osmium tetroxide. After dehydration in a series of ethanol solutions, the cells were embedded in epoxy resin. Ultrathin sections were then stained with uranyl acetate and lead citrate. Finally, mitochondria morphology and MAMs were observed under a transmission electron microscope. The distance between the outer mitochondrial membrane and the smooth endoplasmic reticulum membrane is referred to as the MAMs distance [67], and is indicated by a red arrow. **siRNA knockdown**

Marc-145 cells were transfected with the specified siRNA (100 nM) using Lipofectamine RNAiMAX (Thermo Fisher, 13,778,100) for 24 hours in accordance with the manufacturer’s instructions. All siRNAs were procured from GenePharma (China), and the sequences are listed in S1 Table.

### Confocal microscopy

Marc-145 cells were seeded and cultured in confocal dishes for 12 hours, followed by 24-hour infection with 0.1 MOI of PRRSV with or without concurrent treatment with the specified drugs for 24 hours. Subsequently, the cells were incubated with 150 nM MitoTracker Red CMXRos (Cell Signaling Technology, 9082) for 30 minutes at 37°C. The cells were then washed three times with PBS and permeabilized with 0.1% Triton X-100 for 10 minutes. Next, the cells were blocked with 10% bovine serum albumin (BSA)/PBS for 30 minutes at 37°C and then incubated with primary antibodies in 5% BSA/PBS for 2 hours at 37°C, followed by three washes with PBS buffer under gentle shaking. Finally, the cells were incubated with DAPI for 10 minutes. Throughout the process, the samples were shielded from light.

To observe mitophagy, incubate Marc-145 cells in a confocal dish for 12 hours. Transfect the cells with 1 μg of GFP-LC3 and continue incubation for an additional 12 hours before infecting with PRRSV at an MOI of 0.1. After 24 hours, wash the cells three times with PBS and permeabilize with 0.1% Triton X-100 for 10 minutes. Block the cells with 10% BSA/PBS at 37°C for 30 minutes, then incubate with the primary antibody in 5% BSA/PBS at 37°C for 2 hours. Wash the cells gently three times with PBS, and finally, incubate with DAPI for 10 minutes.

### Western blot analysis

Total cellular protein was extracted using RIPA buffer (20 mM Tris [pH 7.5], 150 mM NaCl, 1% Triton X-100, sodium pyrophosphate, β-glycerophosphate, EDTA, Na_3_VO_4_, and leupeptin). The protein concentrations were then determined by BCA assay, according to the manufacturer’s instructions (Beyotime, China, P0010S). Subsequently, 30 μg of protein, boiled and denatured, was separated by 10% SDS-PAGE and transferred to a PVDF membrane (EMD Millipore, USA). The membranes were blocked with 5% skim milk in TBS-T (50 mM Tris-HCl [pH 7.6], 150 mM NaCl, and 0.1% Tween-20) for 2 hours at room temperature. They were then incubated with the specified primary antibodies overnight at 4°C, followed by horseradish peroxidase-conjugated secondary antibodies for 1 hour at room temperature. The images were developed using Chemistar High-sig ECL western blotting substrate using a Tanon 5200 system (Tanon, China), and the intensities of the western blot bands were analyzed using Image J 1.8.0 software (National Institutes of Health).

### Flow cytometry

Marc-145 cells were seeded and cultured in confocal dishes for 12 hours, followed by 24-hour infection with 0.1 MOI of PRRSV with or without concurrent treatment with the specified drugs for 24 hours. The steps for detecting Ca^2+^ in mitochondria were as follows: cells were incubated in Hanks medium (137.93 mM NaCl, 5.33 mM KCl, 4.17 mM NaHCO_3_, 0.441 mM KH_2_PO_4_, 0.338 mM Na_2_HPO_4_, 5.56 mM D-Glucose) containing 5 μM Rhod-2 (Thermo Fisher Scientific, R1244) at 37°C for 30 minutes. Then, the cells were washed three times with Hanks, followed by incubation at 37°C for 30 minutes. Finally, the samples were analyzed by flow cytometry (CytoFLEX, Beckman Coulter, USA).

The procedure for measuring superoxide levels and the membrane potential in mitochondria involved the following steps: cells were incubated in D-Hanks medium (137.93 mM NaCl, 5.33 mM KCl, 4.17 mM NaHCO_3_, 1.26 mM CaCl_2_, 0.493 mM MgCl_2_, 0.407 mM MgSO_4_, 0.441 mM KH_2_PO_4_, 0.338 mM Na_2_HPO_4_, 5.56 mM D-Glucose) containing 500 nM MitoSOX Red (Thermo Fisher Scientific, M36008) or 1× JC-1 (Thermo Fisher Scientific, T3168) at 37°C for 30 min. Then, the cells were washed three times with D-Hanks. Finally, the cells were analyzed by flow cytometry (CytoFLEX, Beckman Coulter, USA). JC-1 is a dye that enters cells and accumulates in mitochondria. It exists as a monomer or aggregate. In mitochondria with high membrane potential, JC-1 forms aggregates, emitting strong red fluorescence (PE, x-axis). When the membrane potential decreases, JC-1 shifts to the monomer form, producing green fluorescence (FITC, y-axis) [68].

### RNA extraction and quantitative RT-PCR

Collect cell lysates and isolate RNA using the RNA extraction kit (Omega Bio-tek, R6834). Perform reverse transcription with the HiScript II 1st Strand cDNA Synthesis Kit (Vazyme, R211) according to the manufacturer’s instructions. Finally, conduct qPCR using the AceQ qPCR SYBR Green Master Mix (Vazyme, Q111). The primer sequences used are as follows: q-PINK1 (sense strand: 5’-CCT GGA GTG TGA AAC GCT CT-3’, antisense strand: 5’-CTC CCA CCC TCA CCA TTC AC-3’); q-Parkin (sense strand: 5’-GCA TGC ACA TGA AGT GTC CG-3’, antisense strand: 5’-GAC ACT GTG TAT GCT CCC CC-3’).

### Cell HK, LDH, and lactate assay

Culture Marc-145 cells or PAMs for 12 hours, then pre-treat with the appropriate drug for 2 hours (or transfect with siRNA and incubate for an additional 24 hours). Afterward, infect the cells with PRRSV at an MOI of 0.1. Perform measurements according to the manufacturer’s instructions. All kits were sourced from Abbkine, including the following: CheKine Micro Hexokinase (HK) Activity Assay Kit (Abbkine, KTB1123), CheKine Micro Lactate Dehydrogenase (LDH) Assay Kit (Abbkine, KTB1110), and CheKine Micro Lactate Assay Kit (Abbkine, KTB1100).

### Seahorse XF Cell Mito Stress Test and Glycolytic Rate Assay

Marc-145 cells were plated in XFe96 Cell Culture Microplates and incubated overnight. Simultaneously, a sensor cartridge was hydrated in Seahorse XF Calibrant at 37°C in a non-CO_2_ incubator overnight. Before initiating the assay, the cell culture growth medium in the microplate was replaced with warmed assay medium (XF Base Medium with 5.5 mM glucose, 1.0 mM pyruvate and 2 mM L-glutamine). The microplate was then placed in a 37°C non-CO_2_ incubator for 45 minutes to 1 hour, and compounds were loaded into the XFe96 Sensor Cartridge. For the Seahorse XF Real-Time ATP rate test assay (Agilent, 103592–100), the compounds in port A included oligomycin (final concentration: 1.5 μM), and port B contained rotenone (final concentration: 0.5 μM), with antimycin A added for a final concentration of 0.5 μM. In the Seahorse XF Glycolytic Rate Assay (Agilent, 103344–100), the compound in port A consisted of Rot/AA at a final concentration of 0.5 μM, while port B contained 2-DG at a final concentration of 50 mM. Subsequently, the assay was conducted using an Agilent Seahorse XFe96, and the results were analyzed using Wave Desktop and Report Generator software.

### TCID_50_ determination

Marc-145 cells were plated at a density of 1×10^4^ cells per well in a 96-well plate and cultured for 12 hours. Subsequently, the cells were infected with 100 μL of a 10-fold serial dilution of the virus and incubated at 37°C for 2 hours. The viral supernatant was then replaced with DMEM supplemented with 2% FBS, and cellular incubation was extended for an additional 5 days. The cytopathic effect (CPE) of the cells was observed and recorded, and the TCID_50_ was calculated using the Reed and Muench method.

### Cell viability assay

According to the guidelines of the Enhanced Cell Counting Kit-8 (Beyotime, C0041), Marc-145 cells were seeded at a density of 1×10^4^ in a 96-well plate and cultured until adherent. After transfection with siRNA or treatment with drugs, the cells were cultured for an additional 24 hours. Subsequently, 10 μL of Enhanced CCK-8 solution was added to each well, and the plates were incubated for 1 hour in a cell culture incubator. Absorbance was measured at 450 nm. The relative cell viability of the experimental group was expressed as a ratio to the control group.

### Immunoprecipitation

Marc-145 cells were either mock-infected or infected with PRRSV (MOI = 0.1) for 24 hours. Cell lysates were prepared by incubating cells with RIPA lysis buffer (Beyotime, P0013D) on ice for 20 minutes. The lysates were then centrifuged at 12,000 rpm for 10 minutes at 4°C, and the supernatant was collected. Protein A+G Agarose beads (Beyotime, P2055) were added to the supernatant, followed by overnight shaking at 7 rpm at 4°C. The beads were collected by centrifugation at 2,500 rpm at 4°C and washed three times with pre-cooled PBS. Western blot analysis was conducted after RIPA lysis of the beads.

### Proximity ligation assay (PLA)

Marc-145 cells were either mock-infected or infected with PRRSV (MOI = 0.1) for 24 hours. PLA was performed using the Duolink In Situ Detection Reagents Green (Sigma-Aldrich, DUO92014). According to the manufacturer’s instructions, cells were pre-processed with a sequence of fixation, recovery and permeabilization. Marc-145 cells were blocked with Duolink blocking buffer at 37°C for 1 hour, followed by incubation with rabbit anti-Calnexin antibody and mouse anti-Tom20 antibody at 37°C for 2 hours. Subsequently, the cells were treated with pre-diluted anti-rabbit and anti-mouse minus probes at 37°C for 1 hour. Thereafter, Marc-145 cells were consecutively incubated in 1× ligase and 1× polymerase for 30 and 100 minutes, respectively, and then mounted on slides with Duolink In Situ Mounting Medium containing DAPI.

### Mitochondrial fractionation analysis

Marc-145 cells were either mock-infected or infected with PRRSV (MOI = 0.1) for 24 hours. The cells were then harvested, and cytoplasmic and mitochondrial fractions were prepared using a mitochondria isolation kit according to the manufacturer’s instructions (Thermo Fisher Scientific, 89874). The samples were then subjected to analysis by immunoblotting using specific antibodies.

### Statistical analysis

All data were analyzed using GraphPad Prism 7.0 software (GraphPad Software, Inc.) and are presented as means ± SD. Student’s t-test was used to compare differences between two groups for normally distributed data. A one-way ANOVA with Dunnett’s test was used to compare differences among three groups. A two-way ANOVA with Tukey’s or Sidak’s multiple-comparisons test was used to evaluate experiments involving multiple groups. The P values were calculated from three biological replicates unless otherwise indicated in the figure legends. Data were reproduced in independent experiments as indicated in the legends.

## Supporting information

S1 Fig Cell viability of Mdivi-1 and siRNA targeting DRP1 treatment. (A) Marc-145 cells were either untreated or treated with Mdivi-1 (2.5 μM or 5 μM) or DMSO for 24 h, and the relative cell viability was measured. (B) After 24 h of siRNA-DRP1 transfection of Marc-145 cells, cell lysates were collected for western blotting with DRP1 and β-actin antibodies. The intensities of DRP1 bands were analyzed by Image J software. (C) After transfection with specific siRNA for 24 h, the relative viability of Marc-145 cells was assessed. Data are expressed as means ± SD, n = 3 in A and C. The data are representative of results from three independent experiments.
(TIF)

S2 Fig PRRSV infection increases mitochondrial Ca^2+^ levels. (A) After 24 h of PRRSV infection (MOI = 0.1) in Marc-145 cells, mitochondrial Ca^2+^ was stained with Rhod-2 and observed via confocal microscopy. The fluorescence intensity was analyzed using Image J. (B) Marc-145 cells were mock-infected or PRRSV-infected (MOI = 0.1) for 24 h, and mitochondrial Ca^2+^ levels were analyzed by flow cytometry with Rhod-2 staining. Statistical analysis of the proportion of positive cells is shown on the right. (C-H) Marc-145 cells were treated with DMSO or 10 μM of 2-APB, VBIT-12 or MCU-i4 for 24 h. (C, E and G) The relative of cell viability was determined by CCK-8 assay. (D, F and H) The Ca^2+^ level was measured by flow cytometry following Rhod-2 staining. Data are expressed as means ± SD, n = 6 in A or n = 3 in B—H. *P < 0.05, **P < 0.01, and ***P < 0.001. The data are representative of results from three independent experiments.
(TIF)

S3 Fig The knockdown effect and cell viability of siRNA targeting SIGMAR1. (A and B) Marc-145 cells were transfected with si-SIGMAR1 for 24 h. (A) Cell lysates were collected for western blot analysis with antibodies against SIGMAR1 and β-actin. The abundance of SIGMAR1 is expressed as the ratio to β-actin, analyzed by Image J. (B) Determination of the relative cell viability. Data are expressed as means ± SD, n = 3 in B. The data are representative of results from three independent experiments.
(TIF)

S4 Fig PRRSV infection activates the CaMKKβ-AMPK pathway, and CaN is not associated with PRRSV-induced mitochondrial fission. (A) Marc-145 cells were mock-infected or infected with PRRSV for 24 h, and cell lysates were collected for western blot analysis using the specified antibodies. (B and C) Marc-145 cells were mock-infected or PRRSV-infected for 24 h with CaN inhibitor FK506 (10 μM) treatment. (B) Immunoblot analysis was performed using the designated antibodies. (C) The cells were stained with MitoTracker Red, PRRSV-N antibody, and DAPI, followed by confocal microscopy and statistical analysis of mitochondrial length (n = 25 cells). (D-F) Viability measurement of Marc-145 cells treated with DMSO, Compound C (D), STO-609 (E) or FK506 (F) for 24 h. (G and H) Marc-145 cells were transfected with siRNA-PINNK1 (G), or siRNA-Parkin (H) for 24 h, followed by PRRSV infection (MOI = 0.1) for an additional 24 h. Then cell lysates were collected for western blot analysis with indicated antibodies. Data are expressed as means ± SD, n = 25 in C or n = 3 in D, E and F. The levels of phosphorylated DRP1 were normalized to the total DRP1 protein, while the levels of other proteins were normalized to β-actin. The data are representative of results from three independent experiments.
(TIF)

S5 Fig The knockdown effect and cell viability of siRNA targeting DRP1 adaptors. Marc-145 cells were transfected with siRNA-FIS1 (A and E), siRNA-MFF (B and F), siRNA-MiD49 (C and G), or siRNA-MiD51 (D and H) for 24 h. (A-D) Western blot analysis was conducted on cellular lysates using the designated antibodies. The band intensities were assessed using Image J software. (E-H) Determination of the relative cell viability. Data are expressed as means ± SD, n = 3 in E, F, G and H. The data are representative of results from three independent experiments.
(TIF)

S6 Fig The influence of DRP1 adaptors on PRRSV-induced mitochondrial fission and viral replication. (A and B) After mock transfection or transfection with siRNA-NC, siRNA-MFF (A), or siRNA-MiD51 (B) for 24 h, Marc-145 cells were stained with MitoTracker Red, PRRSV-N antibody, and DAPI, and the mitochondria were observed by confocal microscopy. The mitochondrial lengths of 25 cells per group were measured and statistically analyzed for significance. (C and D) Marc-145 cells were mock-infected or infected with PRRSV (MOI = 0.1) for 24 h. Cell lysates were precipitated using anti-MFF (C) or anti-MiD51 (D) antibodies, and the immunoprecipitated proteins, along with whole-cell lysates (WCL), were analyzed using specific antibodies. Anti-Rabbit immunoglobulin G (IgG) antibodies served as a negative control at the endogenous level. (E and F) Marc-145 cells were transfected with si-MFF (E) or si-MiD51 (F) for 24 hours, then mock-infected or infected with PRRSV (MOI = 0.1) for 24 hours. Afterward, cytoplasmic and mitochondrial fractions were isolated, and the collected lysates were analyzed by western blot. (G-J) Marc-145 cells were transfected with siRNA-FIS1 (E), siRNA-MiD49 (F), siRNA-MFF (G), or siRNA-MiD51 (H) for 24 h, followed by infection with 0.1 MOI PRRSV for an additional 24 h. Subsequently, western blot analysis of cell lysates was performed. The ratio of protein to Tom20 or α-tubulin was analyzed by Image J software. Data are expressed as means ± SD, n = 25 in A and B. The data are representative of results from three independent experiments.
(TIF)

S7 Fig PRRSV infection induces mitophagy. (A and B) After 24 h of mock infection or PRRSV infection (0.1 MOI), Marc-145 cells were stained with MitoSOX Red (A) or JC-1 (B) and analyzed for cellular superoxide levels and mitochondrial membrane potential using flow cytometry. The number of positive cells was then calculated. (C) After transfection with GFP-LC3 for 24 h, Marc-145 cells were mock-infected or infected with PRRSV (0.1 MOI) for an additional 24 h. Subsequently, the cells were stained with Tom20 antibody, PRRSV-N antibody and DAPI. The cells were observed by confocal microscopy, and the colocalization of GFP-LC3 was analyzed by Tom20 staining using Image J. Scale bar,10 μm. (D) After 24 h of either mock infection or infection with PRRSV (0.1 MOI), cells were lysed, and the proteins were subjected to immunoblot analysis. The protein levels were quantified by Image J and normalized to β-actin. (E and F) After 24 hours of mock or PRRSV infection (MOI = 0.1) on Marc-145 cells, the mRNA levels of PINK1 (E) and Parkin (F) were assessed by qPCR. (G) Marc-145 cells were either mock-infected or infected with PRRSV (MOI = 0.1) at different time points post-infection. Cell lysates were then subjected to western blot analysis using antibodies against PINK1, Parkin, LC3, Tom20, and β-actin. Data are expressed as means ± SD, n = 3 in A, B, E and F. *p < 0.05; **p < 0.01; ***p < 0.001. All protein levels were quantified using Image J and normalized to β-actin. The data are representative of results from three independent experiments.
(TIF)

S8 Fig The knockdown effect and cell viability of siRNA targeting PINK1 and Parkin. Marc-145 cells were transfected with siRNA-PINK1 or siRNA-Parkin for 24 h. (A and C) The cell lysates were subjected to immunoblot analysis. The band intensities were analyzed using Image J. (B and D) Assessment of Marc-145 cells viability. Cell viability is expressed as a percentage relative to the control group. Data are expressed as means ± SD (n = 3 in B and D). The data are representative of results from three independent experiments.
(TIF)

S9 Fig Mitochondrial Ca^2+^ contributes to PRRSV-induced glycolysis. (A-L) Marc-145 cells were treated or untreated with Mdivi-1 (10 μM), followed by mock infection or infection with PRRSV (MOI = 0.1) for 24 hours. The real-time OCR (A and G) and ECAR (D and J) of the cells were assessed by Seahorse assay. Statistical analysis of the cellular ATP production capacity and basal respiration capacity (B, C, H and I) or glycolytic capacity (E, F, K and L) was conducted. (M and N) Marc-145 cells were mock-infected or infected with PRRSV (MOI = 0.1) for 24 h, and then the levels of HK (M) and LDH (N) in the cells were measured. Data are expressed as means ± SD (n = 3). *p < 0.05; **p < 0.01; ***p < 0.001. The data are representative of results from three independent experiments.
(TIF)

S10 Fig PRRSV infection inhibits OXPHOS and promotes glycolysis in PAMs. (A-H) PAMs were either mock-infected or infected with PRRSV (MOI = 0.1) for 18 hours. The real-time OCR (A) and ECAR (D) of the cells were assessed using the Seahorse assay. Statistical analysis was performed on cellular ATP production capacity and basal respiration capacity (B and C), as well as glycolytic capacity (E and F). (G and H) Determine the levels of HK (G) and LDH (H) in the cells according to the manufacturer’s instructions from Abkkine. Data are expressed as means ± SD (n = 3). *p < 0.05; **p < 0.01; ***p < 0.001. The data are representative of results from three independent experiments.
(TIF)

S1 Table siRNA sequences for knockdown. The table contains the sequence information for all siRNAs used in the study.
(XLSX)

S1 Data Excel spreadsheet containing the underlying numerical data for Figs 1A, 1B, 2B, 2C, 2E, 2F, 3A, 3B, 3D, 4A, 4B, 4D–4F, 4H, 5B–5E, 6B, 6D, 7A, 7B, 8A, 8B, 8G, 8H, 9A–9R, 10A–10G, 10I, 11A, 11C, 11E, 11F, 11J, 11L, 11N, 12A–12I, 12K, S1A, S1C, S2A–S2H, S3B, S4C–S4F, S5E–S5H, S6A, S6B, S7A–S7C, S7E, S7F, S8B, S8D, S9A–S9N, S10A–S10H in separate sheets. (XLSX)

## References

[1] Chen W, Zhao H, Li Y. Mitochondrial dynamics in health and disease: mechanisms and potential targets. Signal transduction and targeted therapy. 2023; 8(1):333. Epub 2023/09/06. doi: 10.1038/s41392-023-01547-9 ; PubMed Central PMCID: PMC10480456. 37669960 PMC10480456

[2] Giacomello M, Pyakurel A, Glytsou C, Scorrano L. The cell biology of mitochondrial membrane dynamics. Nature reviews Molecular cell biology. 2020;21(4):204–24. Epub 2020/02/20. doi: 10.1038/s41580-020-0210-7 . 32071438

[3] James DI, Parone PA, Mattenberger Y, Martinou JC. hFis1, a novel component of the mammalian mitochondrial fission machinery. The Journal of biological chemistry. 2003;278(38):36373–9. Epub 2003/06/05. doi: 10.1074/jbc.M303758200 . 12783892

[4] Otera H, Wang C, Cleland MM, Setoguchi K, Yokota S, Youle RJ, et al. Mff is an essential factor for mitochondrial recruitment of Drp1 during mitochondrial fission in mammalian cells. The Journal of cell biology. 2010;191(6):1141–58. Epub 2010/12/15. doi: 10.1083/jcb.201007152 ; PubMed Central PMCID: PMC3002033. 21149567 PMC3002033

[5] Kalia R, Wang RY, Yusuf A, Thomas PV, Agard DA, Shaw JM, et al. Structural basis of mitochondrial receptor binding and constriction by DRP1. Nature. 2018;558(7710):401–5. Epub 2018/06/15. doi: 10.1038/s41586-018-0211-2 ; PubMed Central PMCID: PMC6120343. 29899447 PMC6120343

[6] Wang W, Wang Y, Long J, Wang J, Haudek SB, Overbeek P, et al. Mitochondrial fission triggered by hyperglycemia is mediated by ROCK1 activation in podocytes and endothelial cells. Cell metabolism. 2012;15(2):186–200. Epub 2012/02/14. doi: 10.1016/j.cmet.2012.01.009 ; PubMed Central PMCID: PMC3278719. 22326220 PMC3278719

[7] Han XJ, Lu YF, Li SA, Kaitsuka T, Sato Y, Tomizawa K, et al. CaM kinase I alpha-induced phosphorylation of Drp1 regulates mitochondrial morphology. The Journal of cell biology. 2008;182(3):573–85. Epub 2008/08/13. doi: 10.1083/jcb.200802164 ; PubMed Central PMCID: PMC2500141. 18695047 PMC2500141

[8] Xu S, Wang P, Zhang H, Gong G, Gutierrez Cortes N, Zhu W, et al. CaMKII induces permeability transition through Drp1 phosphorylation during chronic β-AR stimulation. Nature communications. 2016;7:13189. Epub 2016/10/16. doi: 10.1038/ncomms13189 ; PubMed Central PMCID: PMC5067512. 27739424 PMC5067512

[9] Li Y, Zheng W, Lu Y, Zheng Y, Pan L, Wu X, et al. BNIP3L/NIX-mediated mitophagy: molecular mechanisms and implications for human disease. Cell death & disease. 2021;13(1):14. Epub 2021/12/22. doi: 10.1038/s41419-021-04469-y ; PubMed Central PMCID: PMC8688453 commercial or associative interest that represents a conflict of interest in connection with the work submitted. 34930907 PMC8688453

[10] Trempe JF, Fon EA. Structure and Function of Parkin, PINK1, and DJ-1, the Three Musketeers of Neuroprotection. Frontiers in neurology. 2013;4:38. Epub 2013/04/30. doi: 10.3389/fneur.2013.00038 ; PubMed Central PMCID: PMC3630392. 23626584 PMC3630392

[11] Li Y, Wang Y, Kim E, Beemiller P, Wang CY, Swanson J, et al. Bnip3 mediates the hypoxia-induced inhibition on mammalian target of rapamycin by interacting with Rheb. The Journal of biological chemistry. 2007;282(49):35803–13. Epub 2007/10/12. doi: 10.1074/jbc.M705231200 . 17928295

[12] Liu L, Feng D, Chen G, Chen M, Zheng Q, Song P, et al. Mitochondrial outer-membrane protein FUNDC1 mediates hypoxia-induced mitophagy in mammalian cells. Nature cell biology. 2012;14(2):177–85. Epub 2012/01/24. doi: 10.1038/ncb2422 . 22267086

[13] Giorgi C, Marchi S, Pinton P. The machineries, regulation and cellular functions of mitochondrial calcium. Nature reviews Molecular cell biology. 2018;19(11):713–30. Epub 2018/08/26. doi: 10.1038/s41580-018-0052-8 . 30143745

[14] Zhao H, Pan X. Mitochondrial Ca(2+) and cell cycle regulation. International review of cell and molecular biology. 2021;362:171–207. Epub 2021/07/14. doi: 10.1016/bs.ircmb.2021.02.015 . 34253295

[15] Elesela S, Lukacs NW. Role of Mitochondria in Viral Infections. Life (Basel, Switzerland). 2021;11(3). Epub 2021/04/04. doi: 10.3390/life11030232 ; PubMed Central PMCID: PMC7998235. 33799853 PMC7998235

[16] Kim SJ, Syed GH, Khan M, Chiu WW, Sohail MA, Gish RG, et al. Hepatitis C virus triggers mitochondrial fission and attenuates apoptosis to promote viral persistence. Proceedings of the National Academy of Sciences of the United States of America. 2014;111(17):6413–8. Epub 2014/04/16. doi: 10.1073/pnas.1321114111 ; PubMed Central PMCID: PMC4035934. 24733894 PMC4035934

[17] Pila-Castellanos I, Molino D, McKellar J, Lines L, Da Graca J, Tauziet M, et al. Mitochondrial morphodynamics alteration induced by influenza virus infection as a new antiviral strategy. PLoS pathogens. 2021;17(2):e1009340. Epub 2021/02/18. doi: 10.1371/journal.ppat.1009340 ; PubMed Central PMCID: PMC7920353. 33596274 PMC7920353

[18] Fujioka Y, Tsuda M, Nanbo A, Hattori T, Sasaki J, Sasaki T, et al. A Ca(2+)-dependent signalling circuit regulates influenza A virus internalization and infection. Nature communications. 2013;4:2763. Epub 2014/01/18. doi: 10.1038/ncomms3763 . 24434940

[19] Zhang Q, Hsia SC, Martin-Caraballo M. Regulation of T-type Ca(2+) channel expression by herpes simplex virus-1 infection in sensory-like ND7 cells. Journal of neurovirology. 2017;23(5):657–70. Epub 2017/06/24. doi: 10.1007/s13365-017-0545-9 ; PubMed Central PMCID: PMC5658792. 28639215 PMC5658792

[20] Adams MJ, Lefkowitz EJ, King AMQ, Harrach B, Harrison RL, Knowles NJ, et al. Changes to taxonomy and the International Code of Virus Classification and Nomenclature ratified by the International Committee on Taxonomy of Viruses (2017). Archives of virology. 2017;162(8):2505–38. Epub 2017/04/24. doi: 10.1007/s00705-017-3358-5 . 28434098

[21] Pejsak Z, Stadejek T, Markowska-Daniel I. Clinical signs and economic losses caused by porcine reproductive and respiratory syndrome virus in a large breeding farm. Veterinary microbiology. 1997;55(1–4):317–22. Epub 1997/04/01. doi: 10.1016/s0378-1135(96)01326-0 . 9220628

[22] Lunney JK, Fang Y, Ladinig A, Chen N, Li Y, Rowland B, et al. Porcine Reproductive and Respiratory Syndrome Virus (PRRSV): Pathogenesis and Interaction with the Immune System. Annual review of animal biosciences. 2016;4:129–54. Epub 2015/12/10. doi: 10.1146/annurev-animal-022114-111025 . 26646630

[23] Diao F, Jiang C, Sun Y, Gao Y, Bai J, Nauwynck H, et al. Porcine reproductive and respiratory syndrome virus infection triggers autophagy via ER stress-induced calcium signaling to facilitate virus replication. PLoS pathogens. 2023;19(3):e1011295. Epub 2023/03/28. doi: 10.1371/journal.ppat.1011295 ; PubMed Central PMCID: PMC10079224. 36972295 PMC10079224

[24] Zhang S, Zeng L, Su BQ, Yang GY, Wang J, Ming SL, et al. The glycoprotein 5 of porcine reproductive and respiratory syndrome virus stimulates mitochondrial ROS to facilitate viral replication. mBio. 2023;14(6):e0265123. Epub 2023/12/04. doi: 10.1128/mbio.02651-23 ; PubMed Central PMCID: PMC10746205. 38047681 PMC10746205

[25] Lim S, Lee SY, Seo HH, Ham O, Lee C, Park JH, et al. Regulation of mitochondrial morphology by positive feedback interaction between PKCδ and Drp1 in vascular smooth muscle cell. Journal of cellular biochemistry. 2015;116(4):648–60. Epub 2014/11/18. doi: 10.1002/jcb.25016 . 25399916

[26] Belosludtsev KN, Dubinin MV, Belosludtseva NV, Mironova GD. Mitochondrial Ca2+ Transport: Mechanisms, Molecular Structures, and Role in Cells. Biochemistry Biokhimiia. 2019;84(6):593–607. Epub 2019/06/27. doi: 10.1134/s0006297919060026 . 31238859

[27] Szabadkai G, Bianchi K, Várnai P, De Stefani D, Wieckowski MR, Cavagna D, et al. Chaperone-mediated coupling of endoplasmic reticulum and mitochondrial Ca2+ channels. The Journal of cell biology. 2006;175(6):901–11. Epub 2006/12/21. doi: 10.1083/jcb.200608073 ; PubMed Central PMCID: PMC2064700. 17178908 PMC2064700

[28] Lee S, Min KT. The Interface Between ER and Mitochondria: Molecular Compositions and Functions. Molecules and cells. 2018;41(12):1000–7. Epub 2018/12/29. doi: 10.14348/molcells.2018.0438 ; PubMed Central PMCID: PMC6315321. 30590907 PMC6315321

[29] Hayashi T, Su TP. Sigma-1 receptor chaperones at the ER-mitochondrion interface regulate Ca(2+) signaling and cell survival. Cell. 2007;131(3):596–610. Epub 2007/11/06. doi: 10.1016/j.cell.2007.08.036 . 17981125

[30] Woods A, Dickerson K, Heath R, Hong SP, Momcilovic M, Johnstone SR, et al. Ca2+/calmodulin-dependent protein kinase kinase-beta acts upstream of AMP-activated protein kinase in mammalian cells. Cell metabolism. 2005;2(1):21–33. Epub 2005/08/02. doi: 10.1016/j.cmet.2005.06.005 . 16054096

[31] Toyama EQ, Herzig S, Courchet J, Lewis TL, Jr., Losón OC, Hellberg K, et al. Metabolism. AMP-activated protein kinase mediates mitochondrial fission in response to energy stress. Science (New York, NY). 2016;351(6270):275–81. Epub 2016/01/28. doi: 10.1126/science.aab4138 ; PubMed Central PMCID: PMC4852862. 26816379 PMC4852862

[32] Cereghetti GM, Stangherlin A, Martins de Brito O, Chang CR, Blackstone C, Bernardi P, et al. Dephosphorylation by calcineurin regulates translocation of Drp1 to mitochondria. Proceedings of the National Academy of Sciences of the United States of America. 2008;105(41):15803–8. Epub 2008/10/08. doi: 10.1073/pnas.0808249105 ; PubMed Central PMCID: PMC2572940. 18838687 PMC2572940

[33] Palmer CS, Elgass KD, Parton RG, Osellame LD, Stojanovski D, Ryan MT. Adaptor proteins MiD49 and MiD51 can act independently of Mff and Fis1 in Drp1 recruitment and are specific for mitochondrial fission. The Journal of biological chemistry. 2013;288(38):27584–93. Epub 2013/08/08. doi: 10.1074/jbc.M113.479873 ; PubMed Central PMCID: PMC3779755. 23921378 PMC3779755

[34] Palma FR, Gantner BN, Sakiyama MJ, Kayzuka C, Shukla S, Lacchini R, et al. ROS production by mitochondria: function or dysfunction? Oncogene. 2024;43(5):295–303. Epub 2023/12/12. doi: 10.1038/s41388-023-02907-z . 38081963

[35] Ma K, Chen G, Li W, Kepp O, Zhu Y, Chen Q. Mitophagy, Mitochondrial Homeostasis, and Cell Fate. Frontiers in cell and developmental biology. 2020;8:467. Epub 2020/07/17. doi: 10.3389/fcell.2020.00467 ; PubMed Central PMCID: PMC7326955. 32671064 PMC7326955

[36] Benard G, Bellance N, Jose C, Melser S, Nouette-Gaulain K, Rossignol R. Multi-site control and regulation of mitochondrial energy production. Biochimica et biophysica acta. 2010;1797(6–7):698–709. Epub 2010/03/17. doi: 10.1016/j.bbabio.2010.02.030 . 20226160

[37] Boyman L, Karbowski M, Lederer WJ. Regulation of Mitochondrial ATP Production: Ca(2+) Signaling and Quality Control. Trends in molecular medicine. 2020;26(1):21–39. Epub 2019/11/27. doi: 10.1016/j.molmed.2019.10.007 ; PubMed Central PMCID: PMC7921598. 31767352 PMC7921598

[38] Tarasov AI, Griffiths EJ, Rutter GA. Regulation of ATP production by mitochondrial Ca(2+). Cell calcium. 2012;52(1):28–35. Epub 2012/04/17. doi: 10.1016/j.ceca.2012.03.003 ; PubMed Central PMCID: PMC3396849. 22502861 PMC3396849

[39] Rabinowitz JD, Enerbäck S. Lactate: the ugly duckling of energy metabolism. Nature metabolism. 2020;2(7):566–71. Epub 2020/07/23. doi: 10.1038/s42255-020-0243-4 ; PubMed Central PMCID: PMC7983055. 32694798 PMC7983055

[40] Li X, Yang Y, Zhang B, Lin X, Fu X, An Y, et al. Lactate metabolism in human health and disease. Signal transduction and targeted therapy. 2022;7(1):305. Epub 2022/09/02. doi: 10.1038/s41392-022-01151-3 ; PubMed Central PMCID: PMC9434547. 36050306 PMC9434547

[41] Thyrsted J, Holm CK. Virus-induced metabolic reprogramming and innate sensing hereof by the infected host. Current opinion in biotechnology. 2021;68:44–50. Epub 2020/10/29. doi: 10.1016/j.copbio.2020.10.004 . 33113498

[42] Thai M, Graham NA, Braas D, Nehil M, Komisopoulou E, Kurdistani SK, et al. Adenovirus E4ORF1-induced MYC activation promotes host cell anabolic glucose metabolism and virus replication. Cell metabolism. 2014;19(4):694–701. Epub 2014/04/08. doi: 10.1016/j.cmet.2014.03.009 ; PubMed Central PMCID: PMC4294542. 24703700 PMC4294542

[43] Chaudhari J, Liew CS, Riethoven JM, Sillman S, Vu HLX. Porcine Reproductive and Respiratory Syndrome Virus Infection Upregulates Negative Immune Regulators and T-Cell Exhaustion Markers. Journal of virology. 2021;95(21):e0105221. Epub 2021/08/12. doi: 10.1128/JVI.01052-21 ; PubMed Central PMCID: PMC8513478. 34379512 PMC8513478

[44] Zhou Y, Frey TK, Yang JJ. Viral calciomics: interplays between Ca2+ and virus. Cell calcium. 2009;46(1):1–17. Epub 2009/06/19. doi: 10.1016/j.ceca.2009.05.005 ; PubMed Central PMCID: PMC3449087. 19535138 PMC3449087

[45] Panda S, Behera S, Alam MF, Syed GH. Endoplasmic reticulum & mitochondrial calcium homeostasis: The interplay with viruses. Mitochondrion. 2021;58:227–42. Epub 2021/03/30. doi: 10.1016/j.mito.2021.03.008 ; PubMed Central PMCID: PMC7612732. 33775873 PMC7612732

[46] Oh SJ, Lim BK, Yun J, Shin OS. CVB3-Mediated Mitophagy Plays an Important Role in Viral Replication via Abrogation of Interferon Pathways. Frontiers in cellular and infection microbiology. 2021;11:704494. Epub 2021/07/24. doi: 10.3389/fcimb.2021.704494 ; PubMed Central PMCID: PMC8292102. 34295842 PMC8292102

[47] Barbier V, Lang D, Valois S, Rothman AL, Medin CL. Dengue virus induces mitochondrial elongation through impairment of Drp1-triggered mitochondrial fission. Virology. 2017;500:149–60. Epub 2016/11/07. doi: 10.1016/j.virol.2016.10.022 ; PubMed Central PMCID: PMC5131733. 27816895 PMC5131733

[48] Fang J, Wang H, Lang L, Li H, Li S, Wang K. AMP-activated kinase regulates porcine reproductive and respiratory syndrome virus infection in vitro. Virus genes. 2022;58(2):133–42. Epub 2022/03/08. doi: 10.1007/s11262-022-01888-7 . 35254586

[49] Prudent J, Zunino R, Sugiura A, Mattie S, Shore GC, McBride HM. MAPL SUMOylation of Drp1 Stabilizes an ER/Mitochondrial Platform Required for Cell Death. Molecular cell. 2015;59(6):941–55. Epub 2015/09/20. doi: 10.1016/j.molcel.2015.08.001 . 26384664

[50] Pagliuso A, Cossart P, Stavru F. The ever-growing complexity of the mitochondrial fission machinery. Cellular and molecular life sciences: CMLS. 2018;75(3):355–74. Epub 2017/08/06. doi: 10.1007/s00018-017-2603-0 ; PubMed Central PMCID: PMC5765209. 28779209 PMC5765209

[51] Wang C, Wang Y. The Role and Mechanism of Action of Mitophagy in Various Liver Diseases. Antioxidants & redox signaling. 2023;38(7–9):529–49. Epub 2022/08/27. doi: 10.1089/ars.2022.0114 . 36017629

[52] Su WL, Wu CC, Wu SV, Lee MC, Liao MT, Lu KC, et al. A Review of the Potential Effects of Melatonin in Compromised Mitochondrial Redox Activities in Elderly Patients With COVID-19. Frontiers in nutrition. 2022;9:865321. Epub 2022/07/08. doi: 10.3389/fnut.2022.865321 ; PubMed Central PMCID: PMC9251345. 35795579 PMC9251345

[53] Chen Q, Fang L, Wang D, Wang S, Li P, Li M, et al. Induction of autophagy enhances porcine reproductive and respiratory syndrome virus replication. Virus research. 2012;163(2):650–5. Epub 2011/11/29. doi: 10.1016/j.virusres.2011.11.008 ; PubMed Central PMCID: PMC7114478. 22119900 PMC7114478

[54] Liu Q, Qin Y, Zhou L, Kou Q, Guo X, Ge X, et al. Autophagy sustains the replication of porcine reproductive and respiratory virus in host cells. Virology. 2012;429(2):136–47. Epub 2012/05/09. doi: 10.1016/j.virol.2012.03.022 ; PubMed Central PMCID: PMC7111961. 22564420 PMC7111961

[55] Sun MX, Huang L, Wang R, Yu YL, Li C, Li PP, et al. Porcine reproductive and respiratory syndrome virus induces autophagy to promote virus replication. Autophagy. 2012;8(10):1434–47. Epub 2012/06/29. doi: 10.4161/auto.21159 . 22739997

[56] Zhou Y, Li Y, Tao R, Li J, Fang L, Xiao S. Porcine Reproductive and Respiratory Syndrome Virus nsp5 Induces Incomplete Autophagy by Impairing the Interaction of STX17 and SNAP29. Microbiology spectrum. 2023;11(2):e0438622. Epub 2023/02/24. doi: 10.1128/spectrum.04386-22 ; PubMed Central PMCID: PMC10101144. 36815765 PMC10101144

[57] Li W, Zhang M, Wang Y, Zhao S, Xu P, Cui Z, et al. PRRSV GP5 inhibits the antivirus effects of chaperone-mediated autophagy by targeting LAMP2A. mBio. 2024;15(8):e0053224. Epub 2024/06/28. doi: 10.1128/mbio.00532-24 ; PubMed Central PMCID: PMC11323736. 38940560 PMC11323736

[58] Pinto MC, Kihara AH, Goulart VA, Tonelli FM, Gomes KN, Ulrich H, et al. Calcium signaling and cell proliferation. Cellular signalling. 2015;27(11):2139–49. Epub 2015/08/16. doi: 10.1016/j.cellsig.2015.08.006 . 26275497

[59] Kong F, You H, Zheng K, Tang R, Zheng C. The crosstalk between pattern-recognition receptor signaling and calcium signaling. International journal of biological macromolecules. 2021;192:745–56. Epub 2021/10/12. doi: 10.1016/j.ijbiomac.2021.10.014 . 34634335

[60] Fontaine KA, Sanchez EL, Camarda R, Lagunoff M. Dengue virus induces and requires glycolysis for optimal replication. Journal of virology. 2015;89(4):2358–66. Epub 2014/12/17. doi: 10.1128/JVI.02309-14 ; PubMed Central PMCID: PMC4338897. 25505078 PMC4338897

[61] Ren L, Zhang W, Zhang J, Zhang J, Zhang H, Zhu Y, et al. Influenza A Virus (H1N1) Infection Induces Glycolysis to Facilitate Viral Replication. Virologica Sinica. 2021;36(6):1532–42. Epub 2021/09/15. doi: 10.1007/s12250-021-00433-4 ; PubMed Central PMCID: PMC8692537. 34519916 PMC8692537

[62] Passalacqua KD, Lu J, Goodfellow I, Kolawole AO, Arche JR, Maddox RJ, et al. Glycolysis Is an Intrinsic Factor for Optimal Replication of a Norovirus. mBio. 2019;10(2). Epub 2019/03/14. doi: 10.1128/mBio.02175-18 ; PubMed Central PMCID: PMC6414699. 30862747 PMC6414699

[63] Pang Y, Zhou Y, Wang Y, Fang L, Xiao S. Lactate-lactylation-HSPA6 axis promotes PRRSV replication by impairing IFN-β production. Journal of virology. 2024;98(1):e0167023. Epub 2023/12/13. doi: 10.1128/jvi.01670-23 ; PubMed Central PMCID: PMC10804950. 38088561 PMC10804950

[64] Zhang L, Liu X, Mao J, Sun Y, Gao Y, Bai J, et al. Porcine reproductive and respiratory syndrome virus-mediated lactate facilitates virus replication by targeting MAVS. Veterinary microbiology. 2023;284:109846. Epub 2023/08/17. doi: 10.1016/j.vetmic.2023.109846 . 37586149

[65] Lu Q, Bai J, Zhang L, Liu J, Jiang Z, Michal JJ, et al. Two-dimensional liquid chromatography-tandem mass spectrometry coupled with isobaric tags for relative and absolute quantification (iTRAQ) labeling approach revealed first proteome profiles of pulmonary alveolar macrophages infected with porcine reproductive and respiratory syndrome virus. Journal of proteome research. 2012;11(5):2890–903. Epub 2012/04/11. doi: 10.1021/pr201266z . 22486680

[66] Chen X, Zhang Q, Bai J, Zhao Y, Wang X, Wang H, et al. The Nucleocapsid Protein and Nonstructural Protein 10 of Highly Pathogenic Porcine Reproductive and Respiratory Syndrome Virus Enhance CD83 Production via NF-κB and Sp1 Signaling Pathways. Journal of virology. 2017;91(18). Epub 2017/07/01. doi: 10.1128/jvi.00986-17 ; PubMed Central PMCID: PMC5571251. 28659471 PMC5571251

[67] Giacomello M, Pellegrini L. The coming of age of the mitochondria-ER contact: a matter of thickness. Cell death and differentiation. 2016;23(9):1417–27. Epub 2016/06/25. doi: 10.1038/cdd.2016.52 ; PubMed Central PMCID: PMC5072433. 27341186 PMC5072433

[68] Perelman A, Wachtel C, Cohen M, Haupt S, Shapiro H, Tzur A. JC-1: alternative excitation wavelengths facilitate mitochondrial membrane potential cytometry. Cell death & disease. 2012;3(11):e430. Epub 2012/11/23. doi: 10.1038/cddis.2012.171 ; PubMed Central PMCID: PMC3542606. 23171850 PMC3542606

