# Supplementary figures and images for: Calcium-mediated mitochondrial fission and mitophagy drive glycolysis to facilitate arterivirus proliferation
Source: PLoS Pathog. 2025 Jan 13;21(1):e1012872. doi: 10.1371/journal.ppat.1012872 (PMC11761150; doi:10.1371/journal.ppat.1012872)

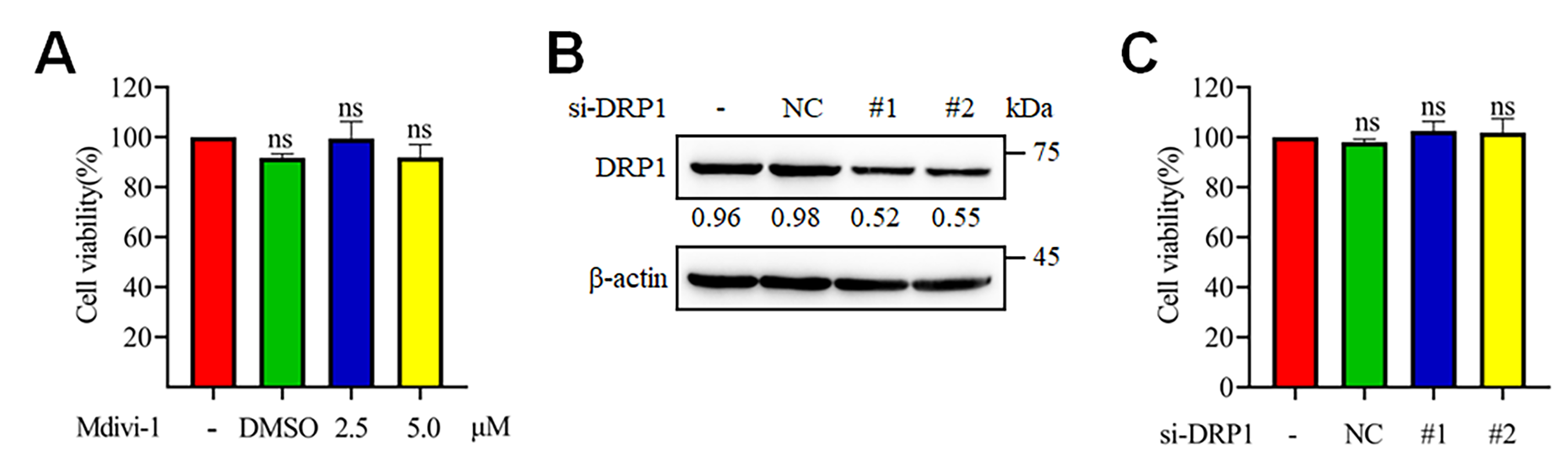

Supplement: S1 Fig — (A) Marc-145 cells were either untreated or treated with Mdivi-1 (2.5 μM or 5 μM) or DMSO for 24 h, and the relative cell viability was measured. (B) After 24 h of siRNA-DRP1 transfection of Marc-145 cells, cell lysates were collected for western blotting with DRP1 and β-actin antibodies. The intensities of DRP1 bands were analyzed by Image J software. (C) After transfection with specific siRNA for 24 h, the relative viability of Marc-145 cells was assessed. Data are expressed as means ± SD, n = 3 in A and C. The data are representative of results from three independent experiments. (TIF) [file ppat.1012872.s001.tif]

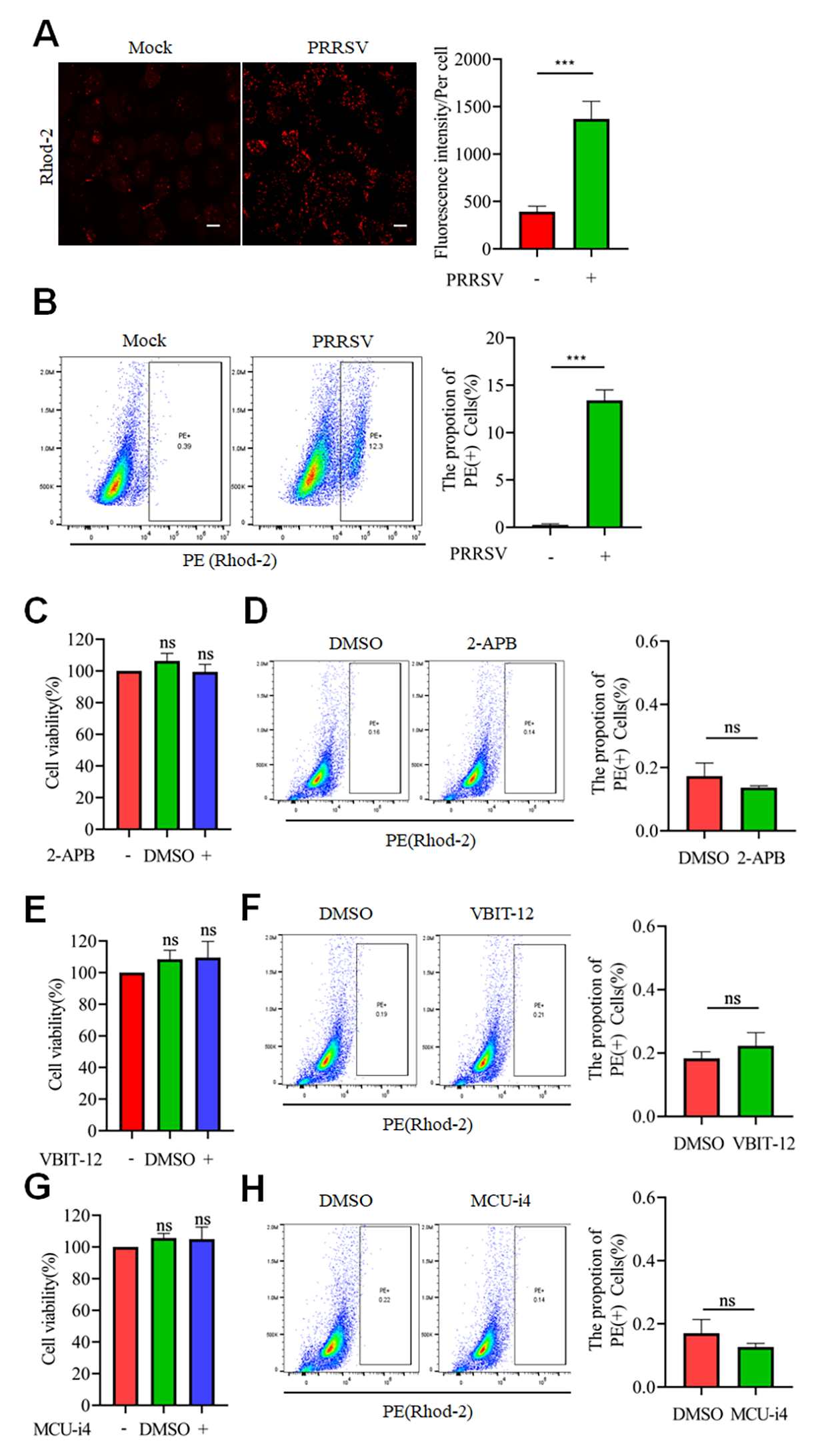

Supplement: S2 Fig — (A) After 24 h of PRRSV infection (MOI = 0.1) in Marc-145 cells, mitochondrial Ca2+ was stained with Rhod-2 and observed via confocal microscopy. The fluorescence intensity was analyzed using Image J. (B) Marc-145 cells were mock-infected or PRRSV-infected (MOI = 0.1) for 24 h, and mitochondrial Ca2+ levels were analyzed by flow cytometry with Rhod-2 staining. Statistical analysis of the proportion of positive cells is shown on the right. (C-H) Marc-145 cells were treated with DMSO or 10 μM of 2-APB, VBIT-12 or MCU-i4 for 24 h. (C, E and G) The relative of cell viability was determined by CCK-8 assay. (D, F and H) The Ca2+ level was measured by flow cytometry following Rhod-2 staining. Data are expressed as means ± SD, n = 6 in A or n = 3 in B—H. *P < 0.05, **P < 0.01, and ***P < 0.001. The data are representative of results from three independent experiments. (TIF) [file ppat.1012872.s002.tif]

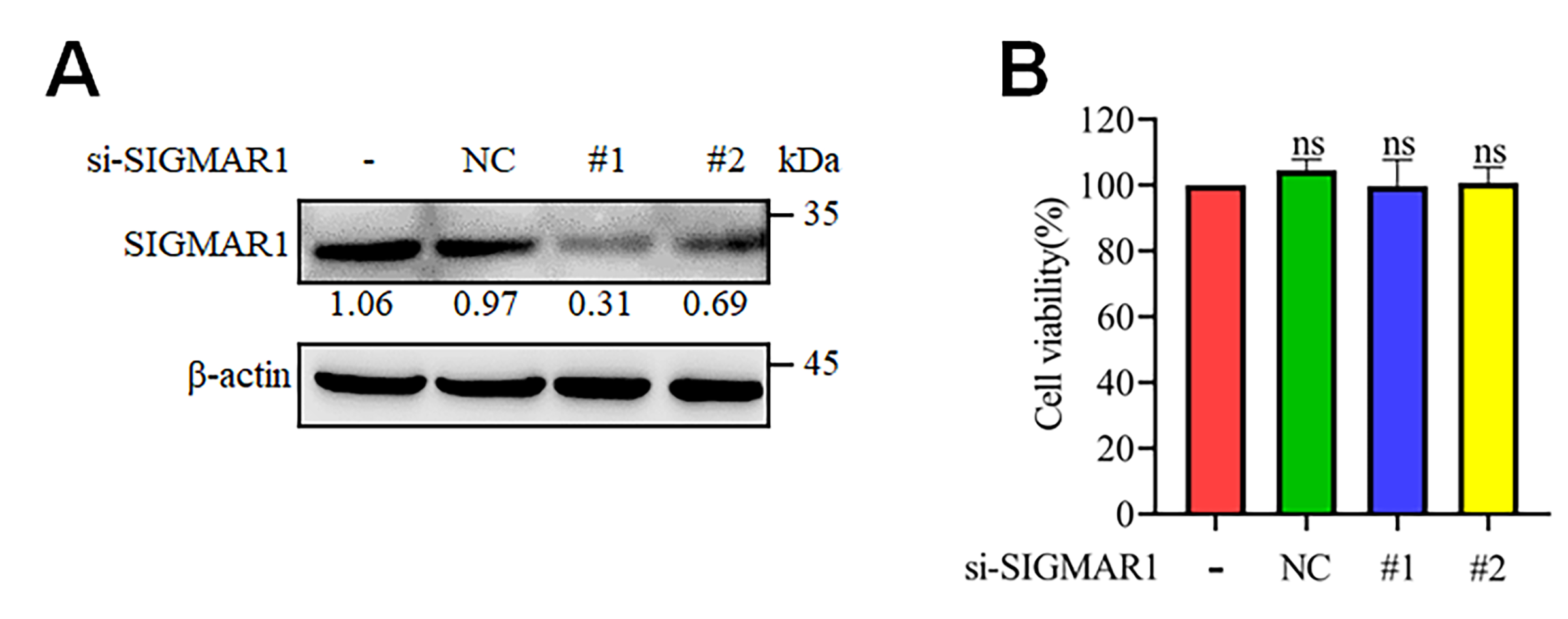

Supplement: S3 Fig — (A and B) Marc-145 cells were transfected with si-SIGMAR1 for 24 h. (A) Cell lysates were collected for western blot analysis with antibodies against SIGMAR1 and β-actin. The abundance of SIGMAR1 is expressed as the ratio to β-actin, analyzed by Image J. (B) Determination of the relative cell viability. Data are expressed as means ± SD, n = 3 in B. The data are representative of results from three independent experiments. (TIF) [file ppat.1012872.s003.tif]

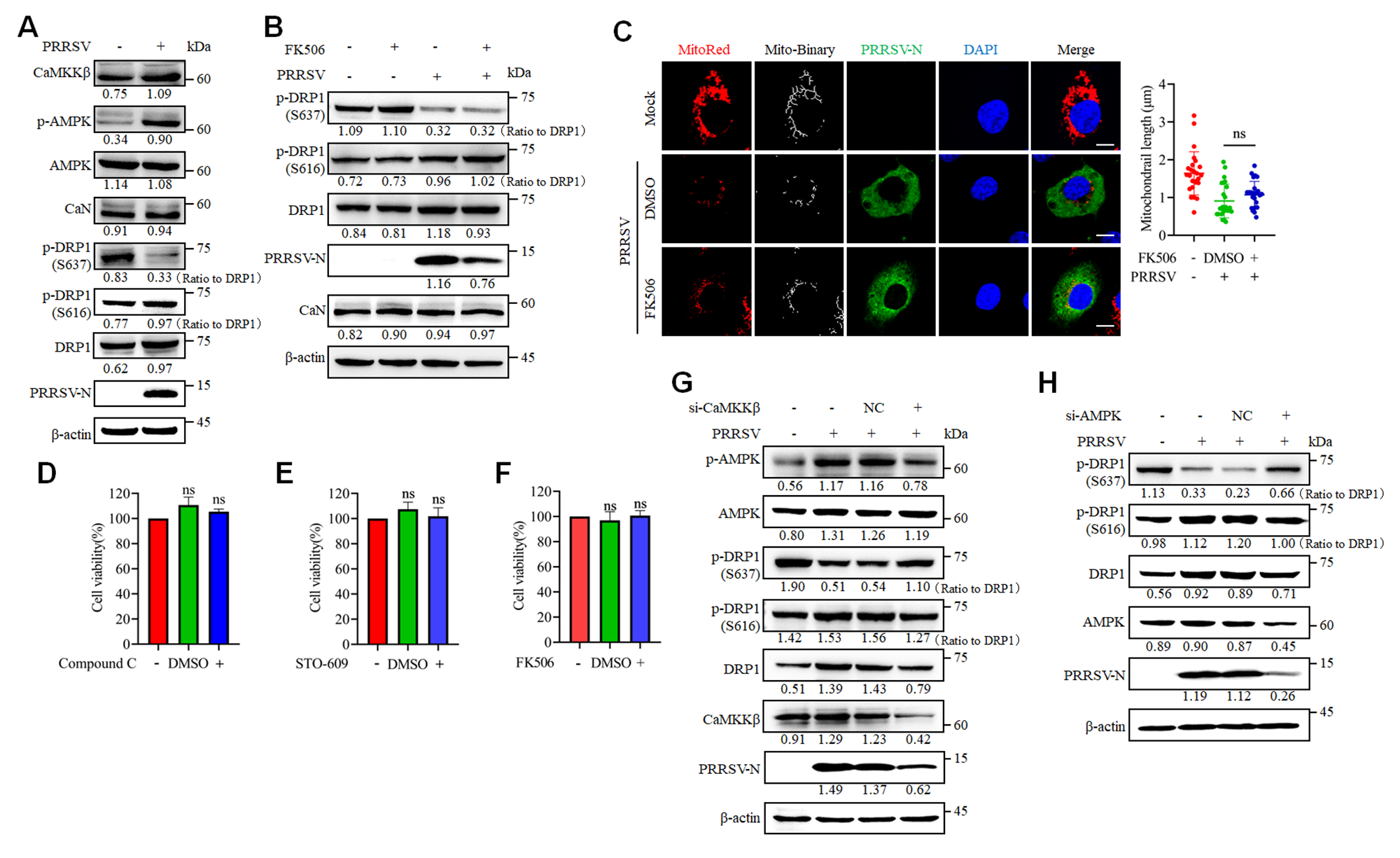

Supplement: S4 Fig — (A) Marc-145 cells were mock-infected or infected with PRRSV for 24 h, and cell lysates were collected for western blot analysis using the specified antibodies. (B and C) Marc-145 cells were mock-infected or PRRSV-infected for 24 h with CaN inhibitor FK506 (10 μM) treatment. (B) Immunoblot analysis was performed using the designated antibodies. (C) The cells were stained with MitoTracker Red, PRRSV-N antibody, and DAPI, followed by confocal microscopy and statistical analysis of mitochondrial length (n = 25 cells). (D-F) Viability measurement of Marc-145 cells treated with DMSO, Compound C (D), STO-609 (E) or FK506 (F) for 24 h. (G and H) Marc-145 cells were transfected with siRNA-PINNK1 (G), or siRNA-Parkin (H) for 24 h, followed by PRRSV infection (MOI = 0.1) for an additional 24 h. Then cell lysates were collected for western blot analysis with indicated antibodies. Data are expressed as means ± SD, n = 25 in C or n = 3 in D, E and F. The levels of phosphorylated DRP1 were normalized to the total DRP1 protein, while the levels of other proteins were normalized to β-actin. The data are representative of results from three independent experiments. (TIF) [file ppat.1012872.s004.tif]

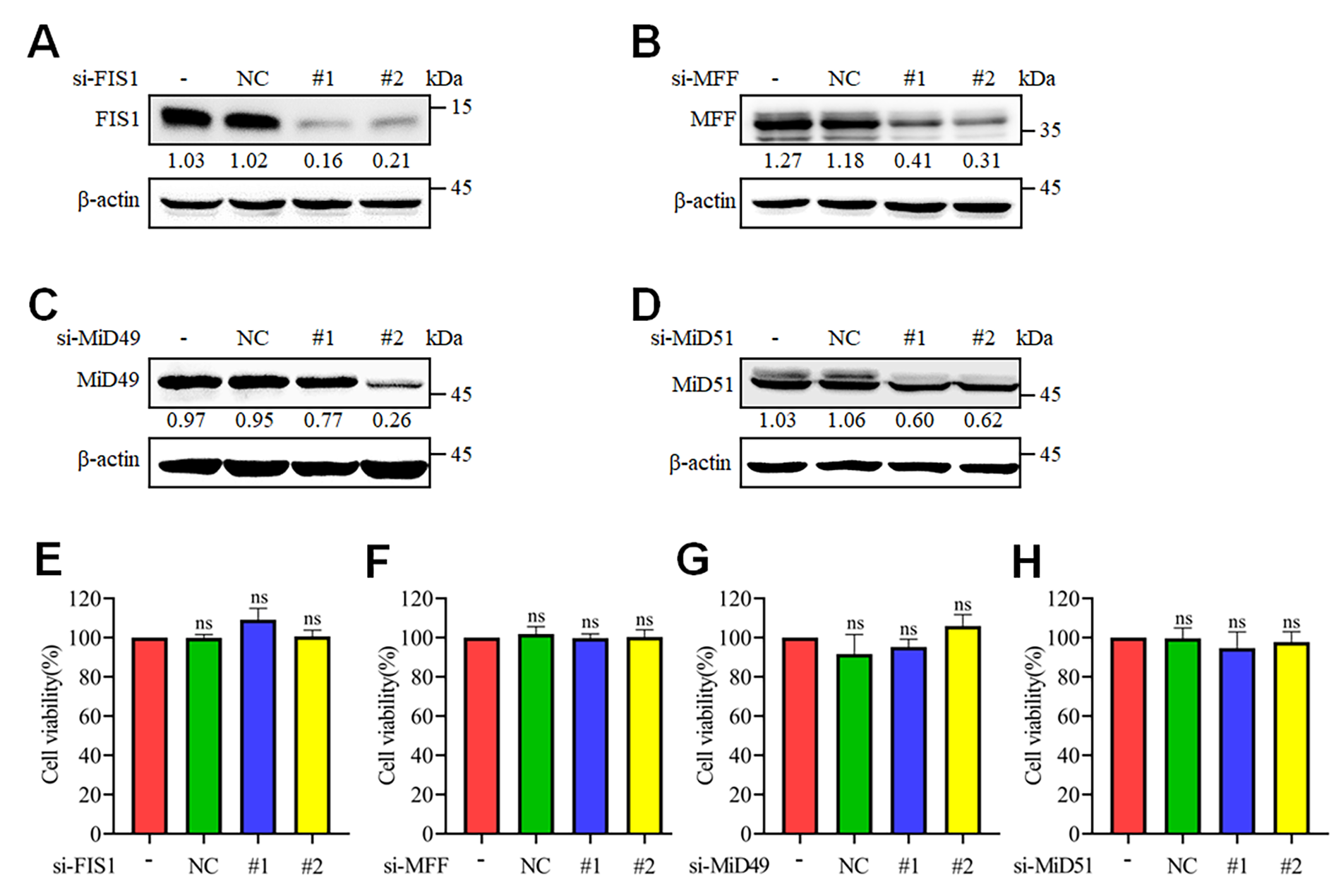

Supplement: S5 Fig — Marc-145 cells were transfected with siRNA-FIS1 (A and E), siRNA-MFF (B and F), siRNA-MiD49 (C and G), or siRNA-MiD51 (D and H) for 24 h. (A-D) Western blot analysis was conducted on cellular lysates using the designated antibodies. The band intensities were assessed using Image J software. (E-H) Determination of the relative cell viability. Data are expressed as means ± SD, n = 3 in E, F, G and H. The data are representative of results from three independent experiments. (TIF) [file ppat.1012872.s005.tif]

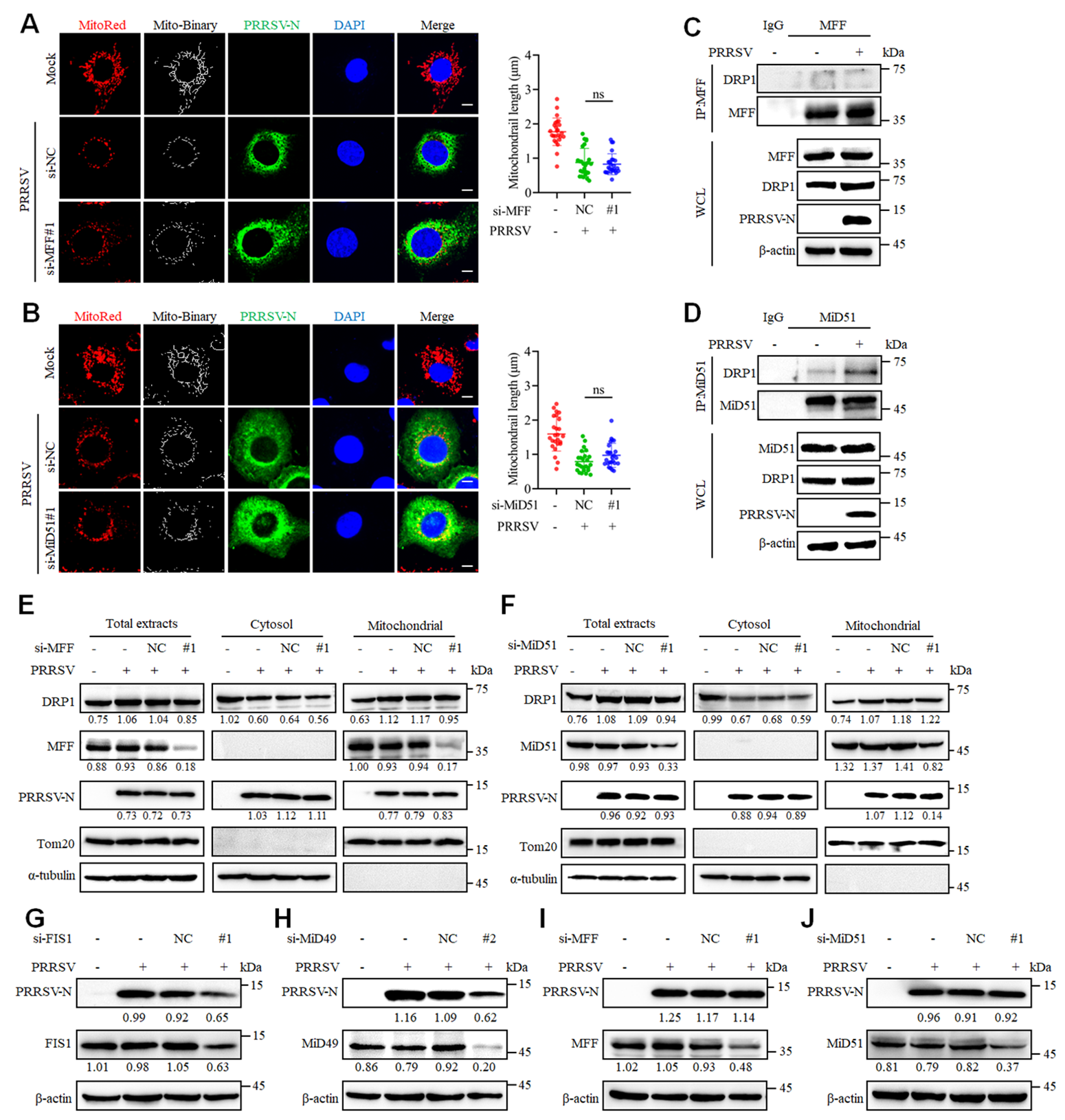

Supplement: S6 Fig — (A and B) After mock transfection or transfection with siRNA-NC, siRNA-MFF (A), or siRNA-MiD51 (B) for 24 h, Marc-145 cells were stained with MitoTracker Red, PRRSV-N antibody, and DAPI, and the mitochondria were observed by confocal microscopy. The mitochondrial lengths of 25 cells per group were measured and statistically analyzed for significance. (C and D) Marc-145 cells were mock-infected or infected with PRRSV (MOI = 0.1) for 24 h. Cell lysates were precipitated using anti-MFF (C) or anti-MiD51 (D) antibodies, and the immunoprecipitated proteins, along with whole-cell lysates (WCL), were analyzed using specific antibodies. Anti-Rabbit immunoglobulin G (IgG) antibodies served as a negative control at the endogenous level. (E and F) Marc-145 cells were transfected with si-MFF (E) or si-MiD51 (F) for 24 hours, then mock-infected or infected with PRRSV (MOI = 0.1) for 24 hours. Afterward, cytoplasmic and mitochondrial fractions were isolated, and the collected lysates were analyzed by western blot. (G-J) Marc-145 cells were transfected with siRNA-FIS1 (E), siRNA-MiD49 (F), siRNA-MFF (G), or siRNA-MiD51 (H) for 24 h, followed by infection with 0.1 MOI PRRSV for an additional 24 h. Subsequently, western blot analysis of cell lysates was performed. The ratio of protein to Tom20 or α-tubulin was analyzed by Image J software. Data are expressed as means ± SD, n = 25 in A and B. The data are representative of results from three independent experiments. (TIF) [file ppat.1012872.s006.tif]

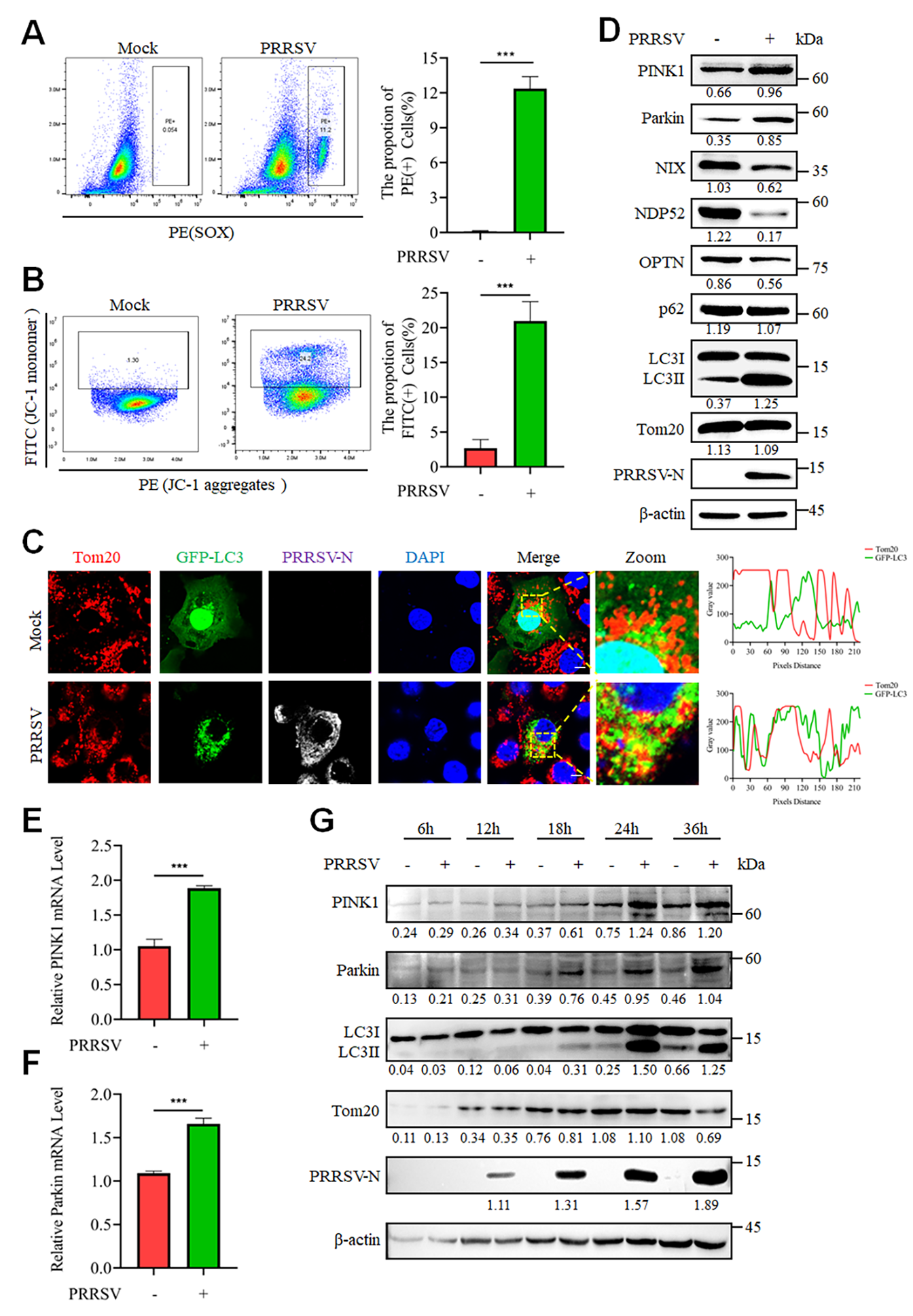

Supplement: S7 Fig — (A and B) After 24 h of mock infection or PRRSV infection (0.1 MOI), Marc-145 cells were stained with MitoSOX Red (A) or JC-1 (B) and analyzed for cellular superoxide levels and mitochondrial membrane potential using flow cytometry. The number of positive cells was then calculated. (C) After transfection with GFP-LC3 for 24 h, Marc-145 cells were mock-infected or infected with PRRSV (0.1 MOI) for an additional 24 h. Subsequently, the cells were stained with Tom20 antibody, PRRSV-N antibody and DAPI. The cells were observed by confocal microscopy, and the colocalization of GFP-LC3 was analyzed by Tom20 staining using Image J. Scale bar,10 μm. (D) After 24 h of either mock infection or infection with PRRSV (0.1 MOI), cells were lysed, and the proteins were subjected to immunoblot analysis. The protein levels were quantified by Image J and normalized to β-actin. (E and F) After 24 hours of mock or PRRSV infection (MOI = 0.1) on Marc-145 cells, the mRNA levels of PINK1 (E) and Parkin (F) were assessed by qPCR. (G) Marc-145 cells were either mock-infected or infected with PRRSV (MOI = 0.1) at different time points post-infection. Cell lysates were then subjected to western blot analysis using antibodies against PINK1, Parkin, LC3, Tom20, and β-actin. Data are expressed as means ± SD, n = 3 in A, B, E and F. *p < 0.05; **p < 0.01; ***p < 0.001. All protein levels were quantified using Image J and normalized to β-actin. The data are representative of results from three independent experiments. (TIF) [file ppat.1012872.s007.tif]

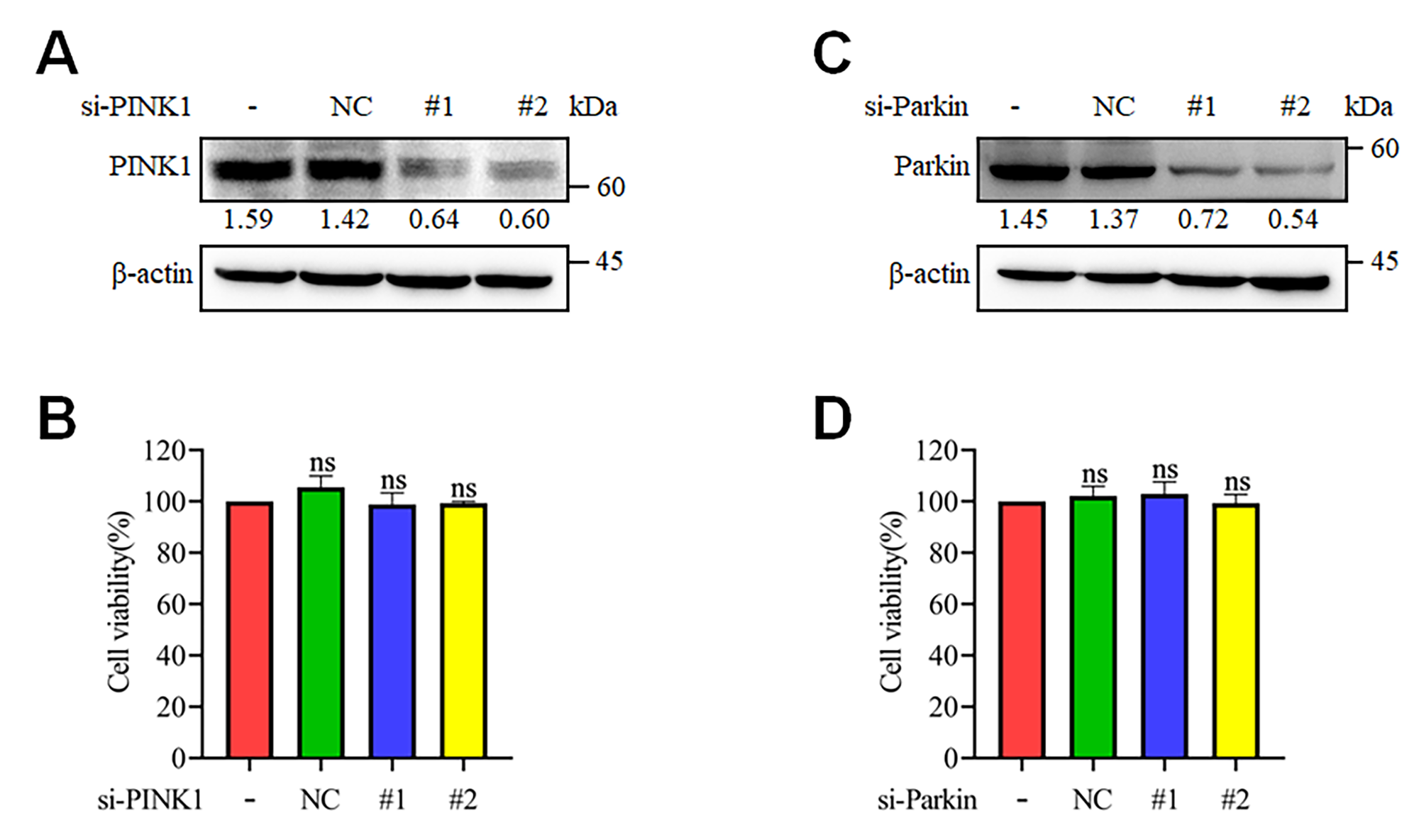

Supplement: S8 Fig — Marc-145 cells were transfected with siRNA-PINK1 or siRNA-Parkin for 24 h. (A and C) The cell lysates were subjected to immunoblot analysis. The band intensities were analyzed using Image J. (B and D) Assessment of Marc-145 cells viability. Cell viability is expressed as a percentage relative to the control group. Data are expressed as means ± SD (n = 3 in B and D). The data are representative of results from three independent experiments. (TIF) [file ppat.1012872.s008.tif]

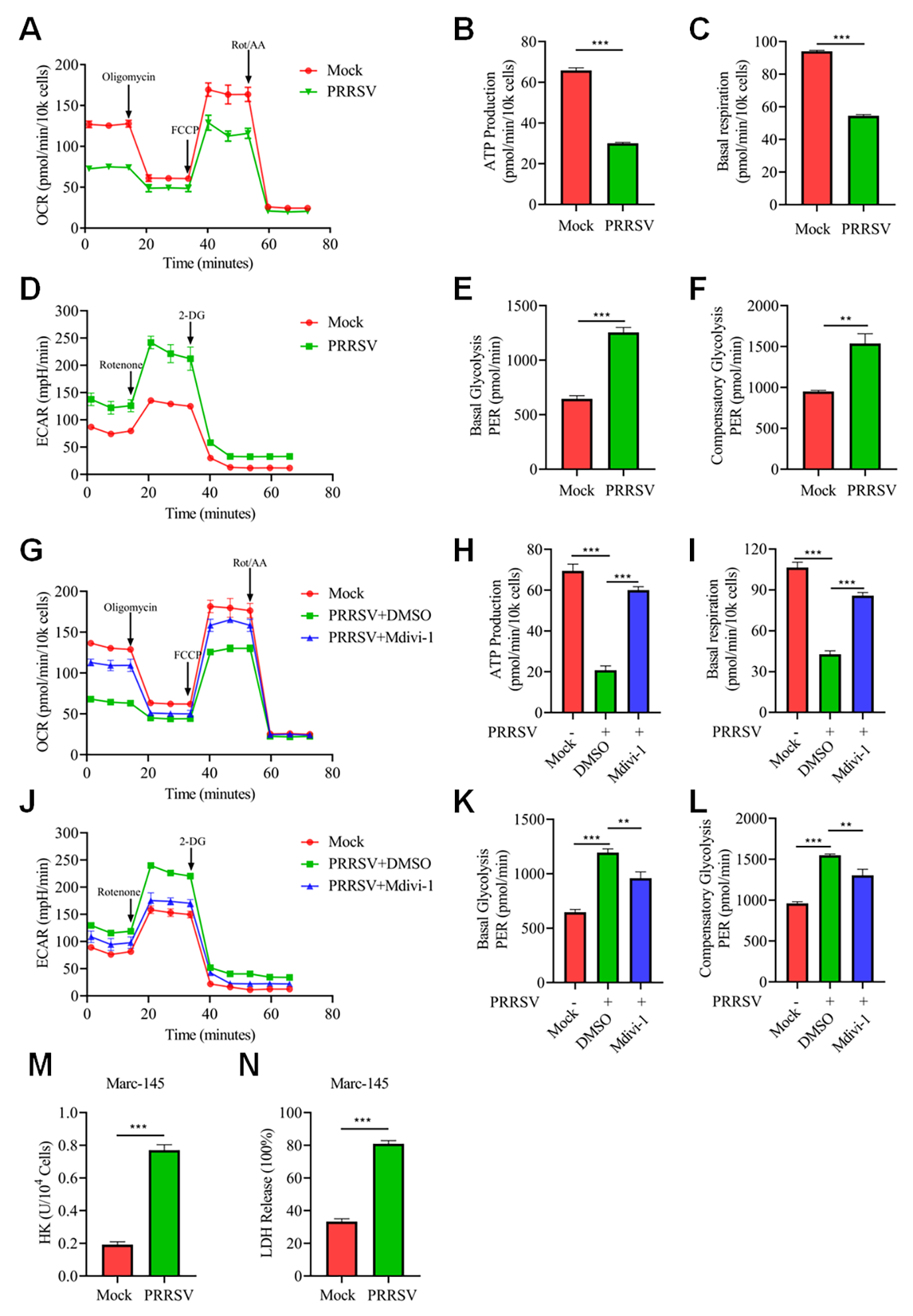

Supplement: S9 Fig — (A-L) Marc-145 cells were treated or untreated with Mdivi-1 (10 μM), followed by mock infection or infection with PRRSV (MOI = 0.1) for 24 hours. The real-time OCR (A and G) and ECAR (D and J) of the cells were assessed by Seahorse assay. Statistical analysis of the cellular ATP production capacity and basal respiration capacity (B, C, H and I) or glycolytic capacity (E, F, K and L) was conducted. (M and N) Marc-145 cells were mock-infected or infected with PRRSV (MOI = 0.1) for 24 h, and then the levels of HK (M) and LDH (N) in the cells were measured. Data are expressed as means ± SD (n = 3). *p < 0.05; **p < 0.01; ***p < 0.001. The data are representative of results from three independent experiments. (TIF) [file ppat.1012872.s009.tif]

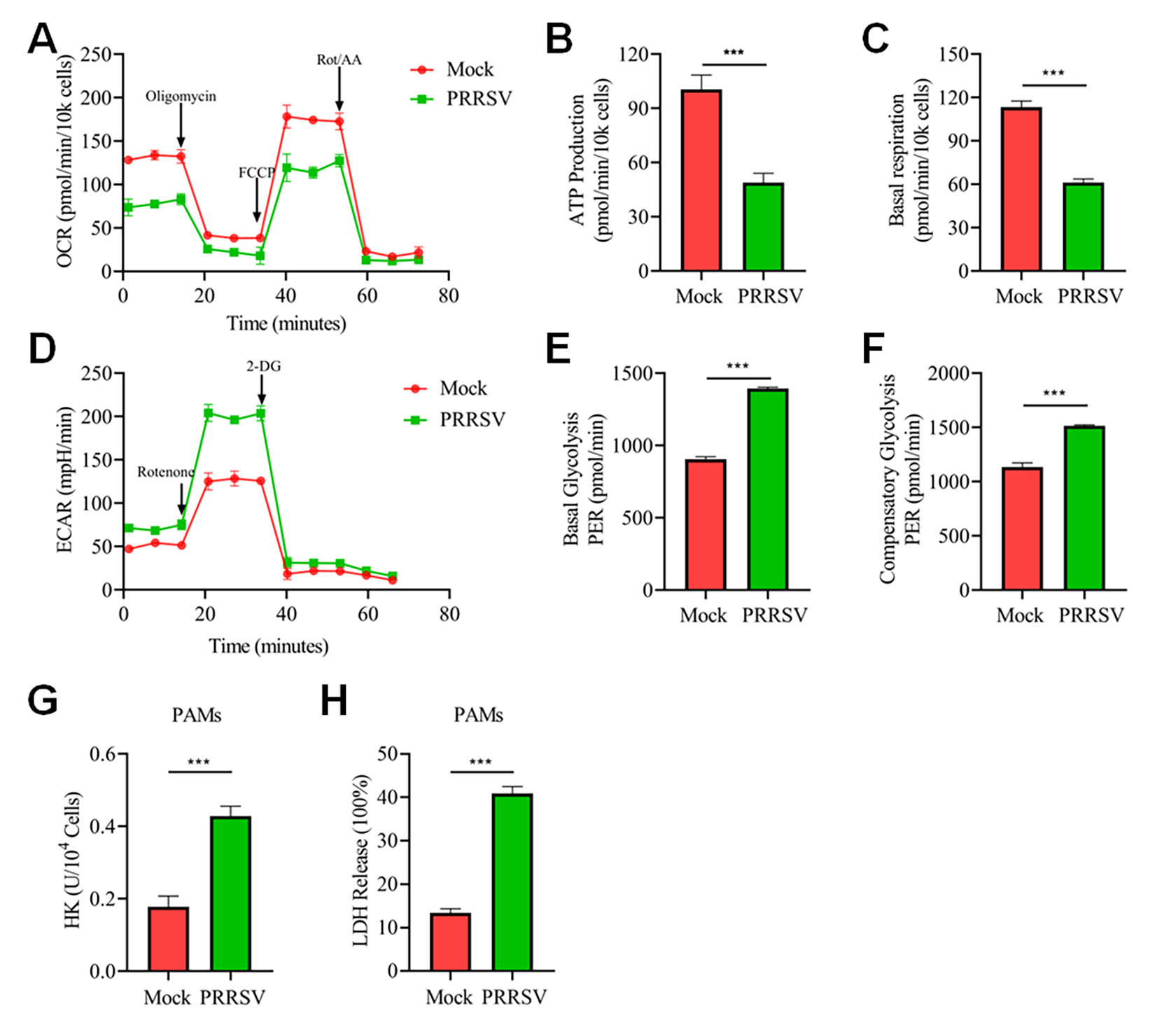

Supplement: S10 Fig — (A-H) PAMs were either mock-infected or infected with PRRSV (MOI = 0.1) for 18 hours. The real-time OCR (A) and ECAR (D) of the cells were assessed using the Seahorse assay. Statistical analysis was performed on cellular ATP production capacity and basal respiration capacity (B and C), as well as glycolytic capacity (E and F). (G and H) Determine the levels of HK (G) and LDH (H) in the cells according to the manufacturer’s instructions from Abkkine. Data are expressed as means ± SD (n = 3). *p < 0.05; **p < 0.01; ***p < 0.001. The data are representative of results from three independent experiments. (TIF) [file ppat.1012872.s010.tif]
